# Diversity, Community Structure, and Antagonism of Endophytic Fungi from Asymptomatic and Symptomatic Mongolian Pine Trees

**DOI:** 10.3390/jof10030212

**Published:** 2024-03-13

**Authors:** Ninghong Ren, Lei Wang, Chongjuan You

**Affiliations:** 1 Beijing Key Laboratory of Forest Pest Control, Beijing Forestry University, Beijing 100083, China; ninghong_ren@bjfu.edu.cn; 2 Honghuaerji Forestry Field, Honghuaerji Forestry Bureau, Hulunbeier 165456, China; lei_wang123@126.com

**Keywords:** Diplodia tip blight, endophytic fungi, high-throughput sequencing, *Pinus sylvestris* var. *mongolica*, antagonistic fungi, community diversity

## Abstract

Diplodia tip blight, caused by *Diplodia sapinea* (=*Sphaeropsis sapinea*), are widely distributed in Honghuaerji, Inner Mongolia, China, causing severe damage on natural Mongolian pine (*Pinus sylvestris* var. *mongolica*). *D. sapinea* is an endophyte that becomes pathogenic under conditions of drought, hail damage, or temperature-associated stress. The role of the endophytic community inhabiting different pine tissues in the expression of disease is still unknown. In this study, the diversity and community structure of endophytic fungi among asymptomatic and symptomatic Mongolian pine were detected using culture-based isolation and high-throughput sequencing (HTS), and the potential antagonistic endophytes against *D. sapinea* were also screened. The results indicated that 198 and 235 strains of endophytic fungi were isolated from different tissues of symptomatic and asymptomatic Mongolian pine, respectively. *D. sapinea* was the most common endophyte isolated from the current-year needles and shoots of symptomatic trees, and *Diplodia* was also the most common in the HTS data. There were no significant differences in the endophytic fungal species richness among asymptomatic and symptomatic trees, but there were differences observed within specific sampled tissues. The ANOSIM analysis confirmed that the endophytic fungi community structure significantly differed between sampling tissues among symptomatic and asymptomatic Mongolian pine. Furthermore, the antagonism study revealed *Penicillium fructuariae-cellae* with the ability to inhibit the growth of *D. sapinea* in vitro, and the potential performance of this fungus, acting as biological control agent, was evaluated under greenhouse. Our findings can pave the way to a better understanding of the interactions between *D. sapinea*, other endophytic fungi and their hosts, and provide helpful information for more efficient disease management strategies.

## 1. Introduction

Mongolian pine (*Pinus sylvestris* var. *mongolica*), a significant pine species indigenous to Asia, has been extensively cultivated in Liaoning, Inner Mongolia, Heilongjiang, and Hebei Provinces of China, due primarily to its rapid growth and exceptional adaptation to cold climates [1]. Since 2018, Diplodia tip blight, caused by *Diplodia sapinea* (Fr.) Fuckel, has been widely distributed in Honghuaerji, Inner Mongolia, China, causing severe damage on natural Mongolian pine. The first evidence of Diplodia tip blight on Mongolian pine was reported in Heilongjiang Province, China in 1980s [2]. This disease is also observed on various Pinus species, especially Scots pine (*Pinus sylvestris*), across parts of Europe such as Germany, Sweden, and Finland, and its incidence has significantly increased over the past decades [3,4,5,6,7,8,9]. Climate change is expected to exacerbate the damage caused by this fungus, as it flourishes in higher temperatures. The reported increase in temperature and drought associated with climate change would also make pine more susceptible to *D. sapinea*, potentially leading to more frequent disease outbreaks. Currently, there is no established and effective method for the disease control.

The pathogen *D. sapinea*, is also known as *Sphaeropsis sapinea* (Fr.: Fr.) Dyko and Sutton. The correct name of this fungus is still in discussion, and the current name in Index Fungorum is *S. sapinea*. The preferred name after the EPPO Global Database, however, is *D. sapinea* [6,8,10] *D. sapinea* is indeed an endophytic fungus that can transition to a pathogenic state under specific environmental conditions, particularly during periods of drought or temperature-associated stress, leading to the development of symptoms, such as tip blight, dieback of current year shoots, stem cankers, blue staining of the sapwood, and ultimately, the decline or death of the pine tree [4,8].

Endophytic fungi are a group of fascinating host-associated fungal communities that live asymptotically inside host tissues for the entire or at least a significant part of their life cycle, without causing apparent negative harm to the host [6,11,12]. It is widely accepted that these fungi have co-evolved with their host trees, and often establish mutualistic relationships that can influence the host fitness [12,13]. The endophytic stage represents a balanced interaction between the fungus and its host. However, endophytic fungi can become pathogen when this balance is disturbed [3,6]. Climate change may influence the lifestyle switch of *D. sapinea*. When climate shifts towards conditions less favorable for host trees (e.g., drought/hailstorm damage), and more conducive for pathogenic fungi (e.g., warmer temperatures), the expression of disease was triggered [4,14,15,16]. The mechanism underlying *D. sapinea*’s transition into a pathogenic state, inducing disease expression, remains incompletely understood.Sherwood et al. [17] proposed that the disease expression may be linked to *D. sapinea*’s ability to exploit metabolites produced by the host in response to stress, such as proline, using these metabolites as a nitrogen source for growth. Furthermore, in order to investigate the role of competitive interactions among endophytes and latent pathogens in determining disease in stressed pine, Oliva et al. [8] concluded that hail promoted *D. sapinea* and other endophytes with a rapid colonization strategy of N-rich substrates. Rapid niche occupation is critical for *D. sapinea* to cause disease after a stress-inducing event. However, competition with other endophytes for key metabolites can suppress the pathogen and prevent trees from developing symptoms.

Despite extensive investigation into the endophytic fungal community of Scots pine, the intricate interactions among different endophytes and their host, as well as with other microbiomes, have yet to be fully elucidated [3,4,6,18]. Bußkamp et al. [4] isolated a total of 103 outgrowing endophytic fungal species from twigs of healthy and diseased Scots pine. Interestingly, the comparison of fungal endophyte communities between twigs from diseased and non-diseased Scots pine trees revealed few differences, except *D. sapinea*, which was the most common endophyte. Blumenstein et al. [6] conducted a comparison of the mycobiome in twigs of healthy and diseased Scots pines using high-throughput sequencing (HTS) of the ITS2 region. They indicated that the mycobiome community composition significantly differed between growth years and sampling time, but not between healthy and diseased trees. The composition of endophytic fungal communities exhibited considerable variation among different host tissues due to differences in microenvironments, nutrient availability, and physiological conditions [19]. Kovalchuk et al. [20] analyzed the mycobiome of different anatomic parts of asymptomatic and symptomatic Norway spruce trees naturally infected by *Heterobasidion*, and they demonstrated that not only does each individual tree tissue (wood, bark, needles, and roots) harbor a unique fungal community, but also that the structure of fungal communities residing in the wood differed significantly among symptomatic and asymptomatic trees. In that sense, differences in the mycobiota among distinct tissues may influence the processes leading to disease outbreak and various disease symptoms. Furthermore, endophytic fungi residing in different tissues of hosts may demonstrate diverse functional traits. For example, root endophytes might directly influence nutrient uptake and root architecture, while leaf endophytes could primarily impact foliar physiology and defense responses [21,22]. The endophytic community, and competition between endophytes can play a role in the Diplodia tip blight disease expression of different tissue (e.g., needles, shoots). Therefore, comprehending the variances in endophytic fungi across diverse host tissues is crucial for deciphering the intricate dynamics of plant-microbe relationships and maximizing their potential advantages in disease control.

The recognition of fungal endophytes’ capacity to enhance host fitness and stress tolerance has sparked the notion of employing these organisms as biocontrol agents [23,24,25]. Studies on conifer trees have demonstrated that inoculations with fungal endophytes can protect the host from natural infections by other pathogens, and these endophytes can be used as antagonists against potential pathogens. Various metabolites with antifungal properties have also been extracted from foliar endophytes of Picea [26,27,28]. The antagonism study revealed 13 possible endophytic fungi with the ability to inhibit the growth of *D. sapinea* in vitro, for example *Sydowia polyspora* (Bref. & Tavel) E. Müll. [18]. Oliva et al. [8] also deliberated on additional endophytic fungi with potential antagonistic properties, such as *Alternaria* sp. and *Epicoccum nigrum* Link.

The objectives of this study were to (i) examine differences in the composition of the endophytic fungal communities associated with various tissues of asymptomatic and symptomatic Mongolian pine, using culture-based isolation and high-throughput sequencing (HTS); (ii) identify the presence of antagonist endophytes in asymptomatic pine; and (iii) screen antagonist endophytes with the ability to inhibit the growth of *D. sapinea* in vitro and under greenhouse inoculation experiment.

## 2. Materials and Methods

### 2.1. Study Sites and Sample Collection

Two natural forest sites of Mongolian pine (Baogentu Forest Farm and Toudaoqiao Forest Farm) in Honghuaerji, Hulunbuir, Inner Mongolia Autonomous Region, China, were chosen for sampling. The two sites are 70 km apart and growing under similar conditions (with a cold temperate continental climate: −1.5 °C–3.7 °C annual mean temperature and 260–490 mm annual mean precipitation, an elevation of 95 m above sea level, and predominantly sandy soils) (https://data.cma.cn/) (accessed on 15 January 2024). The pine stands at both sites were naturally regenerated and approximately 40 years old at the time of sampling.

**Figure 1:**
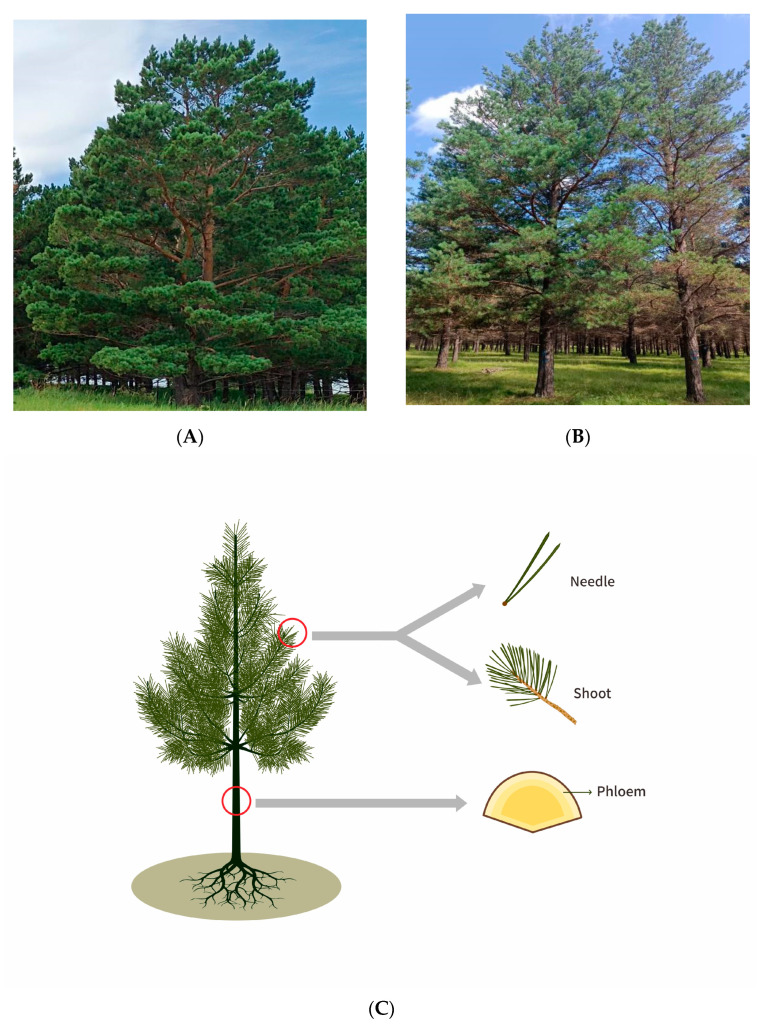
Sampled Mongolian pine trees from the two sampling sites. (**A**) Asymptomatic Mongolian pine from Baogentu Forest Farm; (**B**) symptomatic Mongolian pine from Toudaoqiao Forest Farm; (**C**) schematic diagram illustrating the Mongolian pine tree tissues sampled for analyzing the associated endophytic fungal community.

In July 2022, 30 sampled trees in Baogentu Forest Farm (119°55′57″ E, 48°24′12″ N) (Figure 1A) were classified as ‘asymptomatic’ (<10% crown displaying blight symptoms), while the 30 sampled trees from Toudaoqiao Forest Farm (120°13′38″ E, 48°16′44″ N) (Figure 1B) were considered symptomatic (with 30% or higher percentage of Diplodia tip blight symptoms) [8]. A total of 180 samples were collected, including current-year needles, current-year shoots, and phloem (at breast height) (Figure 1C) from 30 asymptomatic and 30 symptomatic trees. These samples obtained from the current-year needles, current-year shoots, and phloem of asymptomatic trees were labeled as HA, HB, HC, while those from symptomatic trees were labeled as DA, DB, DC. The diameter at breast height of the selected trees ranged from 20 to 25 cm. The samples for culture-based isolation were kept 4 °C and analyzed within 2 days, and the samples for high-throughput sequencing was immediately placed in liquid nitrogen at the site and stored at −80 °C.

### 2.2. Culture-Based Isolation and Molecular Identification

Small sections (5 × 5 mm) were cut from the collected tissue samples, surface-sterilized in 70% ethanol for 1 min, sterilized in 3% (*v*/*v*) sodium hypochlorite solution for 5 min and in 70% ethanol for 30 s, followed by three rinses in sterile distilled water and finally dried on sterilized filter paper [8]. In total, 4–5 cutting segments were then placed on the 0.3% malt extract agar (MEA) media amended with chloramphenicol. All cultures were incubated at 25 °C in the dark for 3 days. Distinct colonies were picked for further purification on 2% MEA media and incubated for seven days [8]. The colony characteristics of each isolate were observed and recorded. One representative per morphotype was used for molecular identification.

Mycelium was scraped from the surface of the colony and DNA was extracted using the cetyltrime thylammonium bromide (CTAB) method [29]. ITS region was amplified using universal ITS primers ITS1/ITS4 [30]. The PCR mixture consisted of 10 μL of 2 × Hieff Master Mix, 7 μL of nucleic acid-free H_2_O, 1 μL of each primer (10 μM), and 1 μL of DNA samples were made up to the final volume of 20 μL. The PCR procedure consisted of an initial denaturation step at 94 °C for 5 min, followed by 35 cycles comprising 30 s at 94 °C, 50 s at 48 °C, and 1 min at 72 °C, with a final elongation step of 7 min at 72 °C. After the PCR amplification, the PCR products were delivered to Beijing Nuosai Genome Research Center Co., Ltd. for sequencing. The DNA sequences were cleaned and blasted in GenBank. The OTU identities were assigned according to match thresholds of >98.5% for species, >97% for genus, >95% for family, >92% for order, >90% for class, and >80% for phylum [8]. The isolated endophytes were accurately identified based on a combination of their morphological characteristics. All isolated strains were stored at 4 °C.

### 2.3. High-Throughput Sequencing

#### 2.3.1. DNA Extraction, Amplification of ITS1 Region, and Sequencing

A total of 60 tissue samples from 10 asymptomatic and 10 symptomatic trees were surface-sterilized, defrosted, and re-sterilized for 1 min in a 3% NaOCl solution. DNA was extracted from 30 mg of the homogenized tissue samples using the TGuide S96 Magnetic Soil /Stool DNA Kit (Tiangen Biotech (Beijing, China) Co., Ltd.) according to the manufacturer’s instructions. The quantification of DNA samples was performed using the Qubit dsDNA HS Assay Kit and Qubit 4.0 Fluorometer (Invitrogen, Thermo Fisher Scientific, Eugene, OR, USA). The primer pair of ITS1F/2 (ITS1:5′-CTTGGTCATTTAGAGGAAGTAA-3′ and ITS2:5′-GCTGCGTTCTTCATCGATGC-3′) was used to amplify the ITS1 region. The PCR was performed in a total reaction volume of 10 μL that included 50 ng of DNA template, 0.3 μL of each primer (10μM), 5 μL of KOD FX Neo buffer, 1 μL of each dNTP2 (2 mM), 0.2 μL of KOD FX Neo, and ddH_2_O to make up the volume to 10 μL. The PCR conditions were as follows: an initial denaturation at 95 °C for 5 min, followed by 25 cycles of denaturation at 95 °C for 30 s, annealing at 50 °C for 30 s, and extension at 72 °C for 40 s, and a final step at 72 °C for 7 min. The PCR products were purified with Agencourt AMPure XP Beads (Beckman Coulter, Indianapolis, IN, USA) and sequenced with Illumina Novaseq 6000 (Illumina, Santiago CA, USA).

#### 2.3.2. Preprocessing and Analysis of ITS1 Sequences

Raw sequences were preprocessed using the BMK Cloud (Biomarker Technologies Co., Ltd., Beijing, China). The raw reads obtained were filtered using Trimmomatic v0.33, and the primer sequences were removed using Cutadapt v1.9.1 to obtain high-quality clean reads. To acquire final valid data (effective reads), sequences from each sample were spliced and length-filtered using Usearch v10, and chimeric sequences were detected and removed using UCHIME v4.2. Clean reads were then denoised using the DADA2 method [31] in QIIME2 [32], which generated amplicon sequence variants (ASVs). Taxonomic analysis of the ASV sequences was performed using a simple Bayesian classifier with UNITE as the reference database. This produced taxonomic data about the species associated with each feature, which subsequently made it possible to analyze the composition of the microbial community at several levels, including phylum, class, order, family, genus, and species.

A rarefaction curve based on sample depth was generated to assess alpha diversity, with ACE, Chao1, Simpson, and Shannon indices employed to gauge species richness and community diversity of endophytic fungi across different tissues of symptomatic and asymptomatic pines. Chao1 and ACE indices were used to estimate species richness, with higher values indicating greater richness. Shannon and Simpson indices were utilized to measure community diversity, where higher values indicate a more diverse community [33]. Beta diversity was assessed to determine the similarity of endophytic fungal communities across samples using QIIME2. Principal coordinate analysis (PCoA) was employed to visualize the endophytic fungal community structure based on Bray–Curtis similarity. PERMANOVA test and analysis of similarities (Anosim) were conducted to determine the significant differences in community structure between different tissues in the pine trees. Additionally, LEfSe analysis was utilized to detect statistically significant differences between various tissues among symptomatic and asymptomatic pines. The linear discriminant analysis (LDA) > 4 and *p* < 0.05 was were considered significantly enriched in that group compared to other groups. FUNGuild was employed to predict the functional profiles of endophytic fungal communities. Endophytes were categorized into three groups based on their nutritional mode: pathotroph (which acquires nutrients by damaging host cells), symbiotroph (which obtains nutrients through resource exchange with host cells), and saprotroph (which obtains nutrients by decomposing deceased host cells).

### 2.4. Antagonism Assay

**Figure 2:**
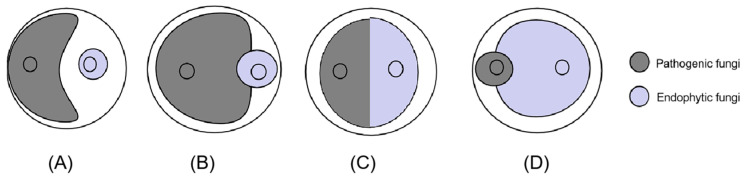
Depiction of the four different reactions during the antagonism assays. (**A**) Inhibition of *D. sapinea* growth; (**B**) endophyte superior; (**C**) neutral or “mutually intermixing growth”; (**D**) *D. sapinea* superior.

**Figure 3:**
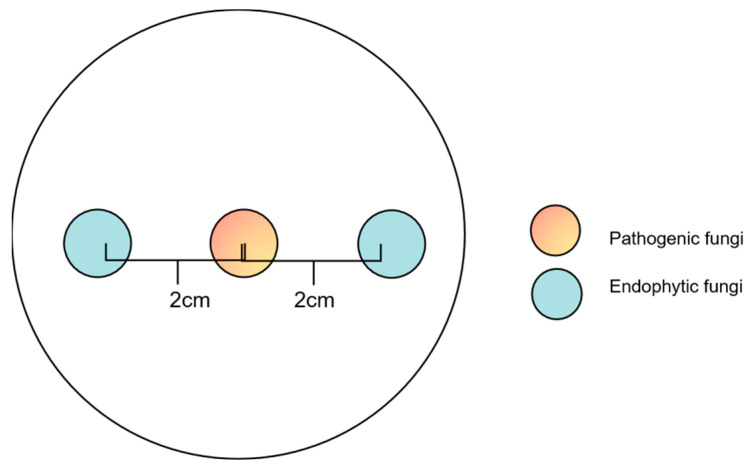
Schematic diagram of a co-culture experiment with preferential placement of endophytic fungi.

**Figure 4:**
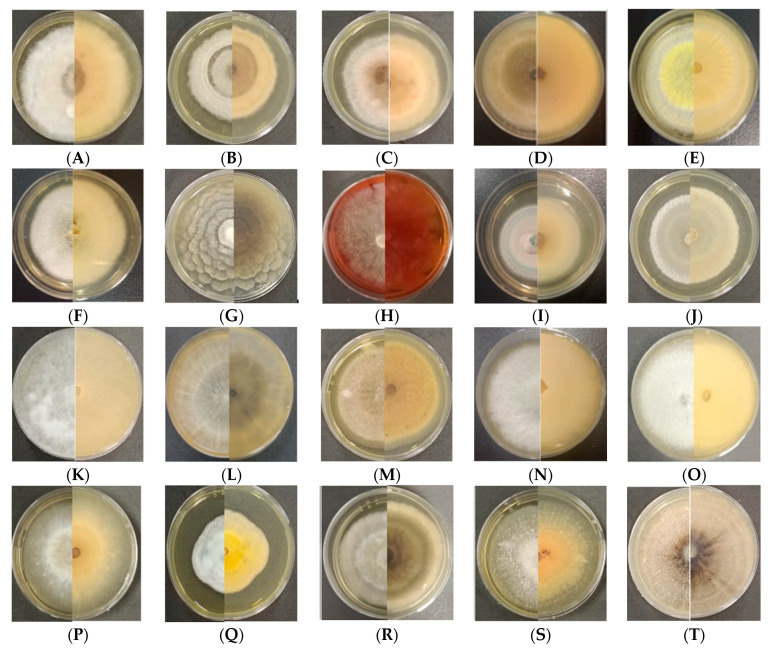
Morphological characteristics of endophytic fungi isolated from different tissues of Mongolian pine (25 °C, seven days in the dark). (**A**) *Alternaria alternata*; (**B**) *Alternaria infectoria*; (**C**) *Alternaria solani*; (**D**) *Alternaria tenuissima*; (**E**) *Aspergillus flavus*; (**F**) *Aspergillus* sp.; (**G**) *Botryotrichum murorum*; (**H**) *Chaetomium globosum*; (**I**) *Cladosporium* sp.; (**J**) *Collariella bostrychodes*; (**K**) *Coprinopsis atramentaria*; (**L**) *Diplodia sapinea*; (**M**) *Dothiorella gregaria*; (**N**) *Fusarium fujikuroi*; (**O**) *Fusarium* sp.; (**P**) *Paracamarosporium hawaiiense*; (**Q**) *Penicillium fructuariae-cella*; (**R**) *Phoma* sp.; (**S**) *Sarocladium zeae*; (**T**)*Sydowia polyspora*.

To identify endophytic fungal isolates with the ability to antagonize the pathogen *D. sapinea*, all endophytic fungal isolates were cultured on MEA at 25 °C for seven days prior to the antagonism experiment in vitro. Mycelial plugs (5 mm in diameter) of the endophytic fungi and the pathogenic fungi *D. sapinea* were placed 4 cm apart on the surface of MEA media [34]. For the control groups (CK), mycelial plugs of *D. sapinea* were placed in the center of MEA media. The Petri dishes were then incubated in the dark at 25 °C. Observations were recorded every 24 h. All treatment experiments were carried out in triplicate. The ability of an endophyte to antagonize the pathogen was determined based on the inhibition level (defined as pathogen growth with and without the endophyte) over a given period of time [18], and it was categorized into four groups: (1) inhibition of *D. sapinea* growth with the presence of a reaction zone (Figure 2A), (2) endophyte superior = the endophyte has overgrown and inhibited mycelial growth (Figure 2B), (3) neutral or ”mutually intermingling growth” = equal mycelial growth capacity between the endophyte and *D. sapinea*, or no apparent mutual inhibition between them (Figure 2C), and (4) *D. sapinea* superior = overgrowth of *D. sapinea* inhibited endophyte mycelial growth (Figure 2D). Growth was assessed 3 and 7 days after inoculation. The inhibition rate was calculated using the formula:

$$\;\mathrm{Inhibition}\;\mathrm{rate}\;=\frac{\left(R1-R2\right)}{R1}\times 100\%\;$$

R1 refers to the radius of the control pathogen colony; R2 refers to the radius of the pathogen colony pointing towards the antagonist fungus.

One-way ANOVA analysis in SPSS 24.0 (IBM Corp., New York, NY, USA) was used to compare the inhibition rates and screen the antagonistic fungi with the highest inhibition rate.

### 2.5. Co-Culture Experiments with Preferential Placement of Endophytic Fungi

After screening for endophytic fungi with the highest inhibition rate, *D. sapinea* and the selected endophytic fungi were cultivated on MEA for three days in the dark at 25 °C. A mycelial plug of the pathogenic fungi *D. sapinea* was placed in the center of the Petri dish, while two mycelial plugs of the selected endophytic fungi were positioned on either side of the Petri dish, 2 cm away from the center (Figure 3). The treatments were as follows: (1) mycelial plugs of the selected endophytic fungi were placed on the MEA medium for 12 h, 24 h, 36 h, and 48 h, followed by the positioning of mycelial plug of *D. sapinea*; (2) simultaneously placed mycelial plugs of the selected endophytic fungi and *D sapinea* onto the MEA medium. For the control group (CK), only mycelial plugs of *D. sapinea* were placed onto the MEA medium. All cultures were then incubated in the dark at 25 °C for seven days. Colony growth was measured on the seventh day, and the inhibition rate of the selected endophyte was calculated using the same formula as mentioned.

### 2.6. Preparation of Antagonistic Endophytic Fungal Fermentation Broth

The endophytic fungus exhibiting the highest inhibition rate against *D. sapinea* was chosen and incubated in the dark at 25 °C for three days. Four mycelial plugs (5 mm in diameter) were then placed in a 250 mL conical flask containing 100 mL of autoclaved MEB liquid media. Fermentation was carried out at 25 °C and 180 rpm on a shaker for 7 days. Following filtration of the fermentation broth through gauze, it was transferred to 2 mL centrifuge tubes and centrifuged at 10,000 rpm for 10 min. 1.5 mL supernatant from each centrifuge tube was removed and filtered through a 0.22 um filter membrane to eliminate mycelium and spores, after which the filtered fermentation broth was stored at 4 °C [35].

### 2.7. Inoculation

Fifteen healthy three-year-old Mongolian pine seedlings were used for the greenhouse inoculation experiment. *D. sapinea* were cultured on 2% MEA and grown at 25 °C for 7 days prior to the experimental inoculations. Wounding inoculations were performed on the current year’s shoots, and the wounds were created using a sterilized needle. Lesion length was recorded daily after the inoculation.

In Group I, the wounded shoots of five pine seedlings were inoculated with mycelial plug (5 mm in diameter) of *D. sapinea*. The mycelium was positioned facing the wound, and the area was sealed with Parafilm to maintain moisture. In Group II, two days after the inoculation of *D. sapinea,* the fermentation broth (5 mL) of the selected antagonistic endophytic fungus, as mentioned in 2.6, was sprayed onto the current-year shoots of five pine seedlings. In Group III (CK), mock-control, the wounded shoots of five pine seedlings were inoculated with sterile MEA plugs (5 mm in diameter).

## 3. Results

### 3.1. Isolation and Identification of Endophytic Fungi from Mongolian Pine

A total of 433 strains were successfully isolated from 2700 tissue segments of 60 pine trees in the 2 sampling plots (Table 1). Among the 235 isolates isolated from the asymptomatic trees, 70 were from needles (HA), 108 from current-year shoots (HB), and 57 from phloem (HC). Meanwhile, the 198 isolates from 30 symptomatic trees included 64 from needles (DA), 86 from shoots (DB), and 48 from phloem (DC). These 433 isolates were initially classified into 57 representative morphotypes according to their cultural characteristics. Based on the ITS sequences similarity, 57 isolates were categorized at the genus level (Table 1; Figure 4). The majority of endophytic fungi were identified as Ascomycota (98.2%), which represented 34 genera. In addition, one genus belonged to Basidiomycota (*Coprinopsis atramentaria)*. The genera of *Sy. polyspora* (22.01%), *D. sapinea* (15.22%), *Cladosporium* sp. (11.48%), and *Penicillium* sp. (10.54%) were most frequently isolated from the asymptomatic and symptomatic Mongolian Pine.

The most abundant species was *Sy. polyspora* (28.51%) in asymptomatic pines, followed by *Cladosporium* sp. (17.45%), and *D. sapinea* (11.49%). *Sy. polyspora* was also most commonly isolated from the current-year needles and shoots of asymptomatic pines, with 45.71% and 23.15%, respectively. *Cladosporium* sp., on the other hand, was most frequently found in the phloem (24.56%) of asymptomatic pines. In symptomatic pines, the most abundant species was *D. sapinea* (19.19%), followed by *Sy. polyspora* (13.64%), and *Penicillium* sp. (11.62%). Within the symptomatic shoots, *D. sapinea* was the most abundant, with a frequency of 25.00%. *D. sapinea* and *Penicillium* sp. were both isolated at a frequency of 15.63%, from the needles of symptomatic trees. In the phloem of symptomatic trees, *Talaromyces pseudofuniculosus* was the most frequently encountered at 18.75%, with *D. sapinea* following at a frequency of 16.67%.

### 3.2. HTS Analysis of Endophytic Fungal Community of Mongolian Pine

#### 3.2.1. Analysis of Endophytic Fungal Community Composition of Mongolian Pine

**Figure 5:**
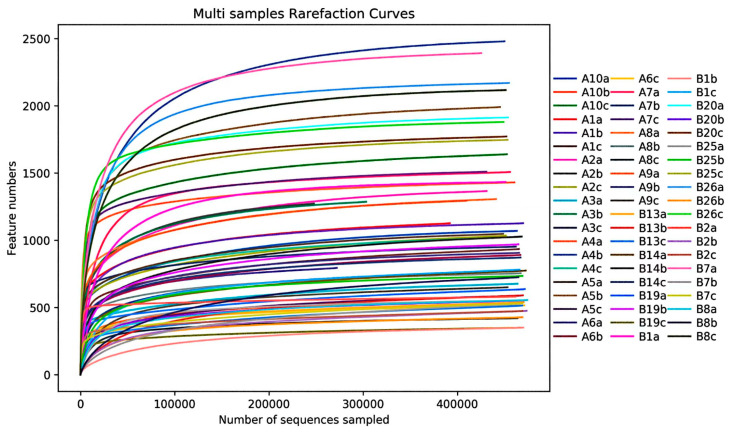
Rarefaction curves for all analyzed samples.

A total of 28,022,878 raw reads were generated from 60 samples, and 27,872,009 clean reads were obtained after data cleaning. Each sample yielded a minimum of 253,437 clean reads and an average of 464,533 clean reads. The rarefaction curves reached stability with increasing sequencing volume, suggesting that the generated sequencing data adequately represent the community composition and structure of endophytic fungi in various tissues of both asymptomatic and symptomatic pines (Figure 5).

**Figure 6:**
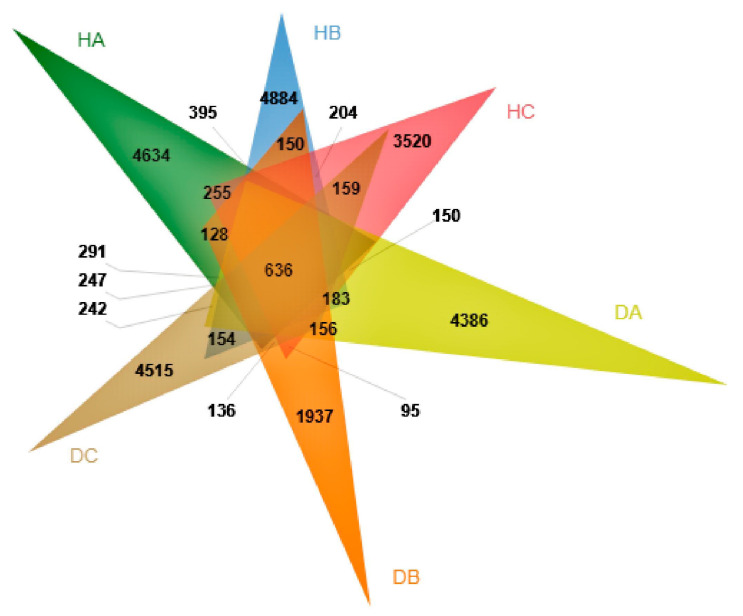
Venn diagram displaying the ASVs shared by different tissues of asymptomatic and symptomatic pines.

The reads were assigned to 30,130 amplicon sequence variants (ASVs). The sampled tissues of the pine trees shared 636 (2.11%) of the total ASVs (Figure 6). The proportion of the ASVs unique to a certain tissue ranged from 0.06% (1937 ASVs; DB) to 16.20% (4884 ASVs; HB), with the shoots of asymptomatic pine trees exhibiting the highest number of specific ASVs. According to the taxonomy annotation, these ASVs were classified into 17 phyla, 60 classes, 158 orders, 383 families, 954 genera, and 1707 species.

**Figure 7:**
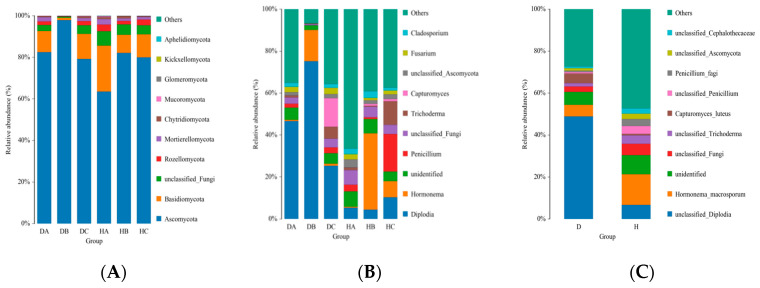
Relative abundances of the endophytic fungal community at phylum (**A**) and genus (**B**) levels in needles, shoots, and phloem of Mongolian pine; relative abundances of the endophytic fungal community at genus (**C**) levels in symptomatic and asymptomatic trees. (H: samples from asymptomatic pine trees, and D: samples from symptomatic trees).

At the phylum level, Ascomycota and Basidiomycota were the predominant constituents of the endophytic fungal communities in various tissues of both asymptomatic and symptomatic pines (Figure 7A). The relative abundances of Ascomycota in DA, DB, DC, HA, HB, and HC were 83%, 98%, 79%, 64%, 82%, and 80%, respectively. Following Ascomycota, Basidiomycota had relative abundances of 12%, 22%, and 11% in DC, HA, and HC, respectively, while it accounted for less than 10% in DA, DB, and HB. Relative abundances of other phyla were below 10%.

Symptomatic and asymptomatic pines exhibited different dominant endophytic fungal taxa at the genus level. *Diplodia* abundance was statistically highest (48.88%) in symptomatic pines, followed by Others (27.28%). While in asymptomatic pines, the “Others” fungi had the highest relative abundance (47.30%), followed by *Hormonema* (14.63%) (Figure 7C). The endophytic fungal communities in various tissues of symptomatic and asymptomatic pines displayed different dominant taxa (Figure 7B). In symptomatic pines, *Diplodia* had higher relative abundance of 46.67% and 74.24% in current-year needles (DA) and shoots (DB), respectively. In the phloem (DC), the “Others” fungi had the highest relative abundance (49.80%), followed by *Diplodia* (25.39%). However, the “Others” was the most abundant in all three tissues of asymptomatic pines (HA: 66.52%, HB: 39.17%, and HC: 39.17%). But the abundance of *Diplodia* was statistically higher in the phloem (10.38%) compared to the other two tissues (HA: 5.33%, HB: 4.47%).

#### 3.2.2. Alpha Diversity of Endophytic Fungal Community in Mongolian Pine

**Figure 8:**
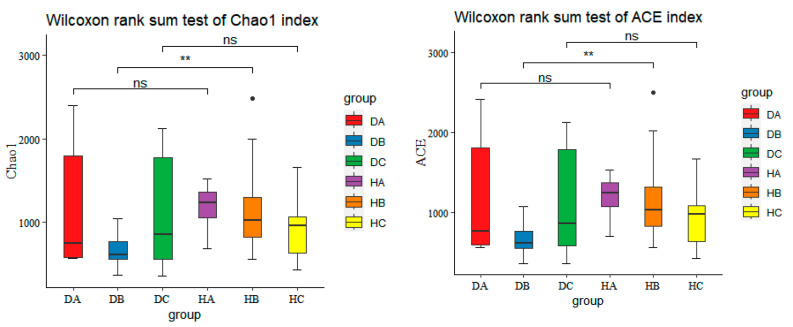
Boxplots of ACE and Chao1 indices (** *p* < 0.01; ‘ns’ represents no significant difference).

**Figure 9:**
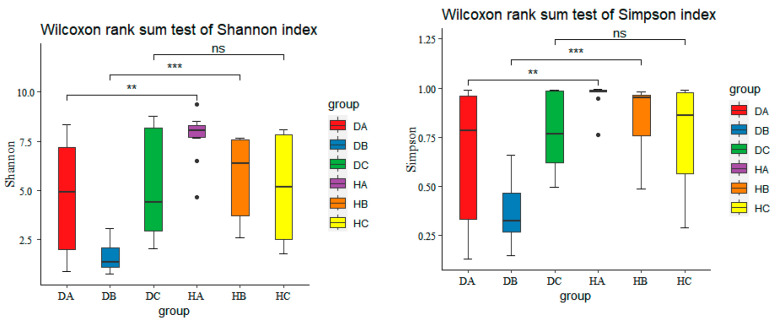
Boxplots of Simpson and Shannon indices (** *p* < 0.01; *** *p* < 0.001; ‘ns’ represents no significant difference).

Alpha diversity was assessed using the ACE and Chao1 indices (Figure 8) to measure species richness and the Simpson and Shannon indices (Figure 9) to evaluate community diversity. The highest richness of endophytic fungal communities in asymptomatic and symptomatic trees were observed in current-year needles (HA) and in phloem (DC), respectively, with the current-year shoots in symptomatic (DB) and the phloem in asymptomatic trees (HC) having the lowest numbers of ASVs, respectively (Figure 8). There were significant differences observed in the species richness of endophytic fungi between shoots of asymptomatic and symptomatic pine trees (HB vs. DB). However, no significant differences were found in the species richness of endophytic fungi among asymptomatic and symptomatic trees in needles and phloem (HA vs. DA and HC vs. DC).

Needles had the highest community diversity in both symptomatic and asymptomatic trees. The lowest community diversity was observed in shoots of symptomatic trees (DB) and in phloem of asymptomatic trees (HC), respectively (Figure 9). There was statistically significant difference in endophytic fungal diversity among asymptomatic and symptomatic trees found in current-year needles and shoots, but no significant differences in phloem of asymptomatic and symptomatic trees (HC vs. DC).

#### 3.2.3. Beta Diversity of Endophytic Fungal Community in Mongolian Pine

**Figure 10:**
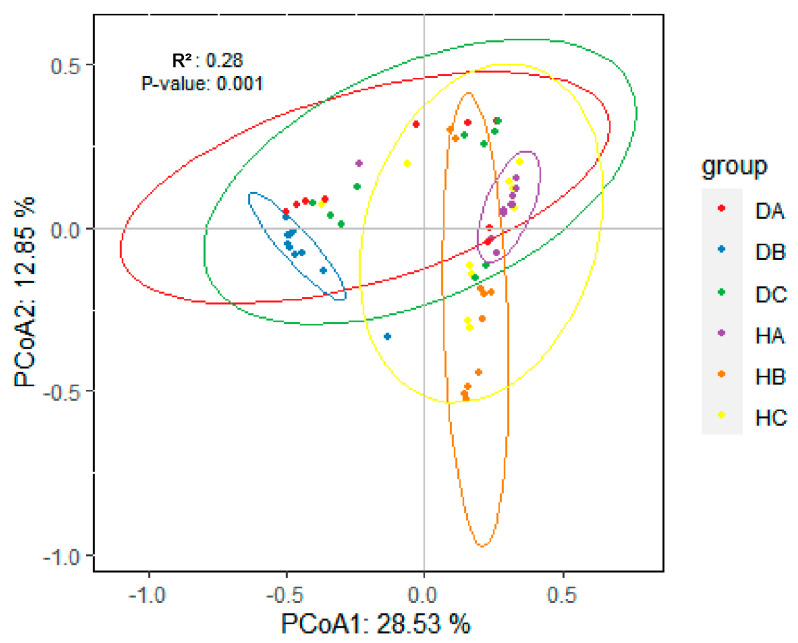
PCoA based on the relative abundance of fungal ASVs, showing the differences in fungal community structure in sampled tissues.

Beta diversity was analyzed using Principal Coordinate Analysis (PCoA), with statistical significance determined using ANOSIM. The PCoA, based on the relative abundance of ASVs, accounted for 41.38% of the observed variation (Figure 10). Significant differences were found in the endophytic fungal community structure between asymptomatic and symptomatic trees (H vs. D) (Figure S1). Additionally, ANOSIM analysis revealed significant differences in the endophytic fungal community structure among the sampled pine tissues (A vs. B vs. C) (*p*-value = 0.001) (Figure S2). Notably, there were significant differences related to needles and shoots among asymptomatic and symptomatic trees (HB vs. DB; HA vs. DA), but no distinction was observed between HC and DC (R^2^ = 0.08, *p*-value = 0.115) (Table 2).

#### 3.2.4. LEfSe Analysis of Endophytic Fungal Community in the Shoots of Asymptomatic and Symptomatic Mongolian Pine

**Figure 11:**
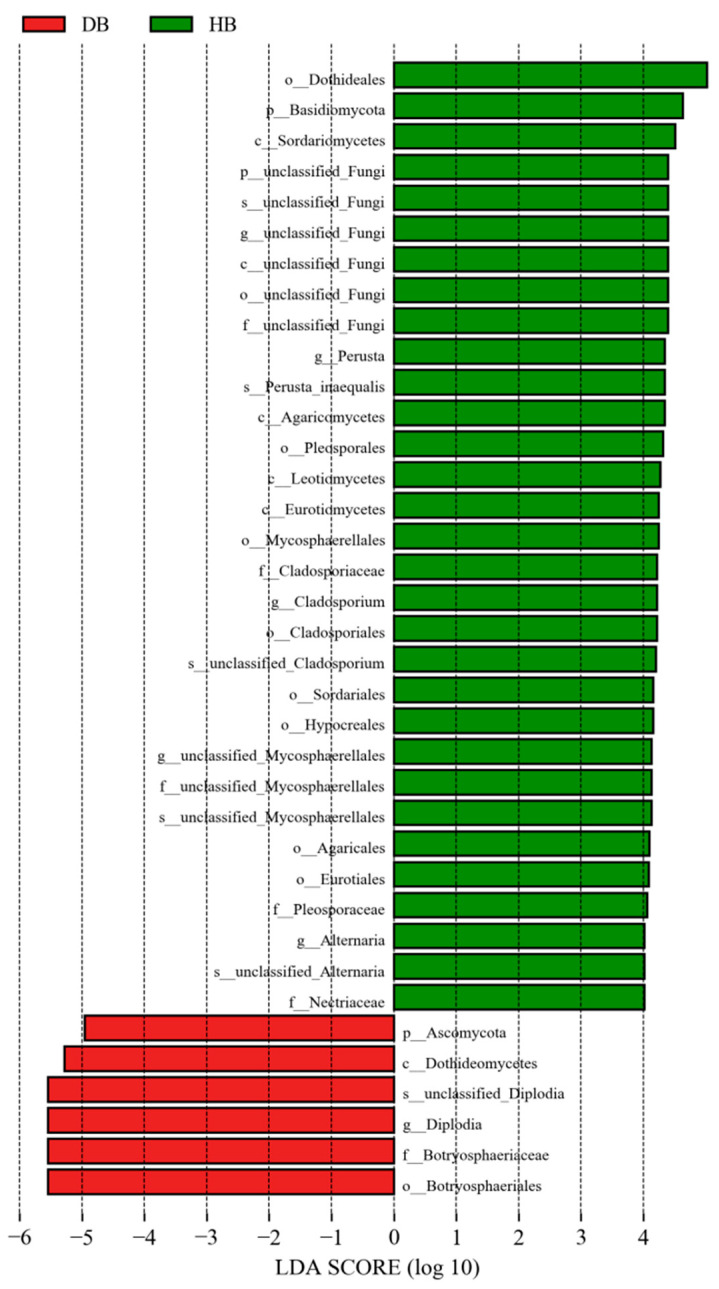
LEfSe rank plots of shoots among asymptomatic and symptomatic Mongolian Pine.

To identify the taxonomic fungi with significantly different abundances among shoots of asymptomatic and symptomatic Mongolian Pine, LEfSe analysis was used for biomarker analysis (LDA score > 4, *p* value < 0.05). As shown in Figure 11, *Diplodia* was representative genus (higher relative abundance) in the shoots of symptomatic pine, while *Perusta*, *Cladosporium*, and *Alternaria* were the significant genus in the shoots of asymptomatic pine.

#### 3.2.5. Functional Prediction of Endophytic Fungal Community in Mongolian Pine

**Figure 12:**
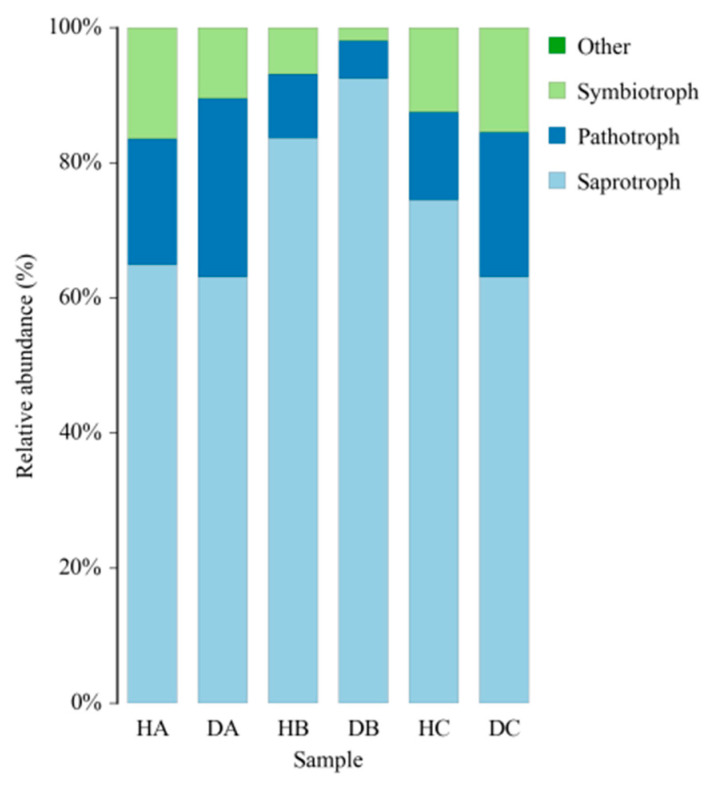
FUNGuild analysis.

**Figure 13:**
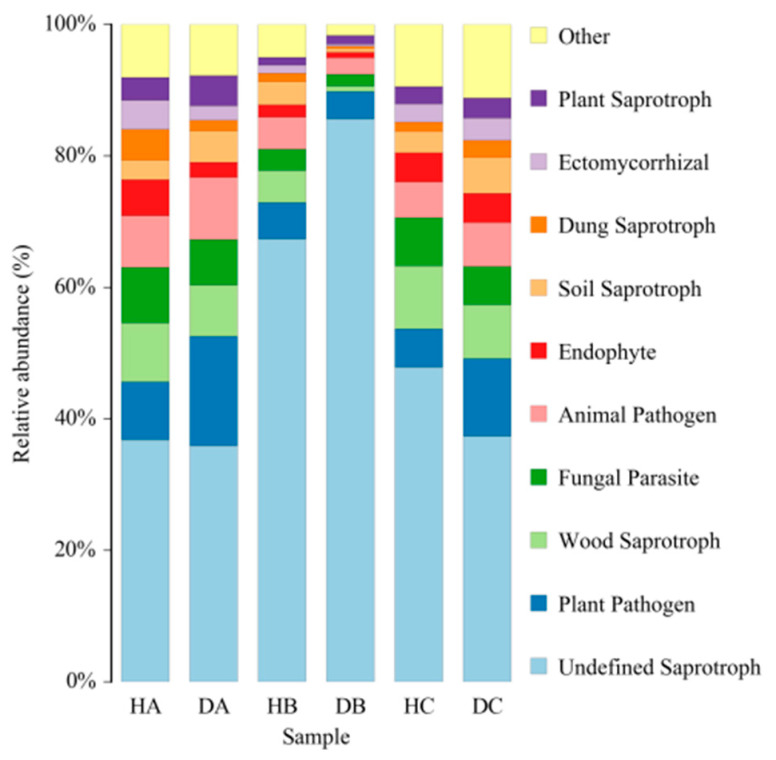
FUNGuild analysis of ASVs from the three tissues of Mongolian pine. The top ten fungi with the highest percentage have been shown.

FUNGuild was used to assess the ecological and functional status of 30,130 ASVs obtained from the sampled trees, of which 12,870 ASVs (42.7%) were successfully defined. Across various tissues of both asymptomatic and symptomatic pines, saprotrophs constituted the largest proportion, followed by pathotrophs and symbiotrophs (Figure 12). The proportion of ASVs classified as saprotrophs within a certain tissue ranged from 63.15% (DA) to 92.49% (DB) (Figure 12). Additionally, Undefined saprotrophs exhibited the highest prevalence in all tissue samples (Figure 13).

### 3.3. In Vitro Study: Antagonism Assay

**Figure 14:**
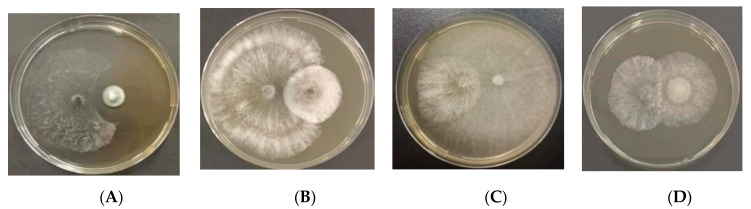
Four different kinds of interaction between the endophytic fungi (on the right) and *D. sapinea* (on the left). (**A**) *D. sapinea* vs. *Pe. fructuariae-cellae;* (**B**) *D. sapinea* vs. *Alternaria alternata;* (**C**) *D. sapinea* vs. *Trichoderma* sp.; (**D**) *D. sapinea* vs. *Phoma* sp.

**Figure 15:**
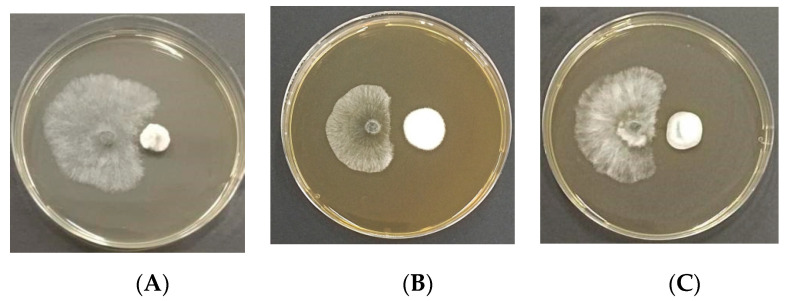
Dual culture of the selected endophytic fungi (on the right) with *D. sapinea* (on the left), showing variations in the diameter of the inhibition zone. (**A**) *D. sapinea* vs. *Taifanglania* sp.; (**B**) *D. sapinea* vs. *Pa. hawaiiense*; (**C**) *D. sapinea* vs. *Pe. fructuariae-cellae*.

Fifty-seven endophytes isolated from the sampled pine trees were employed in the antagonism assay (Table 3). Three isolated endophytes *(Penicillium fructuariae-cellae*, *Taifanglania* sp., and *Paracamarosporium hawaiiense*) inhibited the growth of *D. sapinea* (Figure 14A and Figure 15). Two isolated endophytes (*Aspergillus flavus* and *Phoma* sp.) displayed similar growth capability (Figure 14B), while four isolated endophytes (*Aspergillus* sp., *Tricharina* sp., *Trichoderma afroharzianum*, and *Trichoderma harzianum*) exhibited superior growth over *D. sapinea* (Figure 14C). The remaining tested endophytes showed inferior growth against *D. sapinea* (Figure 14D).

Three selected antagonistic fungi, *Taifanglania* sp., *Pa. hawaiiense*, and *Pe. fructuariae-cellae*, were tested further. *Taifanglania* sp. was isolated from DA, while *Pa. hawaiiense* originated from HB, and *Pe. fructuariae-cellae* was obtained from HB, HC, DB, and DC. All three strains exhibited inhibition rates of over 60% on the mycelial growth of *D. sapinea* (Table 4). *Pe. fructuariae-cellae* (EMD32) and *Pa. Hawaiiense* (EMD29) demonstrated notably higher inhibition rates against *D. sapinea* compared to *Taifanglania* sp. (EMD48). However, the inhibition rates of *Pe. fructuariae-cellae* and *Pa. Hawaiiense* against *D. sapinea* did not show significant differences. Overall, *Pe. fructuariae-cellae* emerged as the most effective endophytic fungus in inhibiting the pathogen *D. sapinea,* and was selected for further analysis.

### 3.4. Co-Culture Experiment with Preferential Placement of Pe. fructuariae-cellae

**Figure 16:**
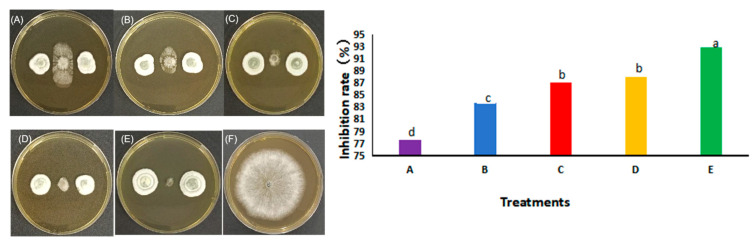
The inhibition rate of *Pe. fructuariae-cellae* against *D. sapinea* at different time periods. (**A**) Concurrently culturing of *Pe. fructuariae-cellae* and *D. sapinea*; (**B**–**E**) preferential culturing of *Pe. fructuariae-cellae* for 12 h (**B**), 24 h (**C**), 36 h (**D**), and 48 h (**E**), followed by co-culturing with *D. sapinea*; (**F**) *D. sapinea* alone. Different lower case letter indicate significant differences (*p* < 0.05).

*Pe. fructuariae-cellae* was initially cultured on MEA medium, after which *D. sapinea* was added to the culture after 12, 24, 36, and 48 h. The findings demonstrated that *Pe. fructuariae-cellae* consistently inhibited *D. sapinea* by over 70% at each tested time point. There was no notable variance in the inhibition rates at 24 and 36 h against *D. sapinea*. The 48-h experiment exhibited the most substantial inhibitory effect, with an inhibition rate of 92.86% (Figure 16).

### 3.5. Greenhouse Inoculation Experiment

**Figure 17:**
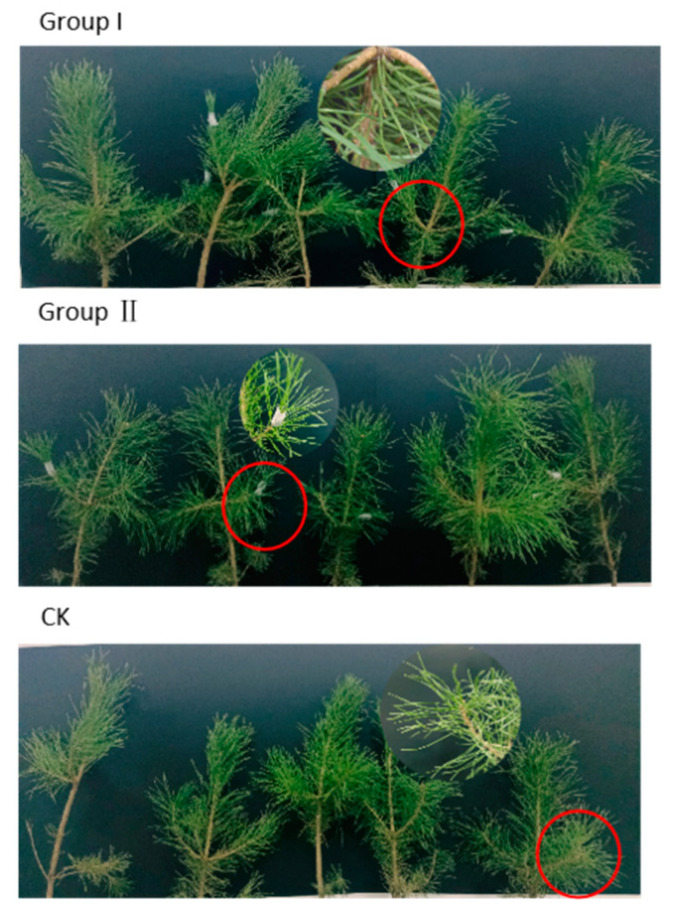
The infected Mongolian pine seedlings on the 7th day after inoculation (The red circle in group I shows the necrosis on the inoculated shoot infected with *D. sapinea*).

*Pe. fructuariae-cellae* (EMD32) was chosen for the greenhouse inoculation experiment. The mock-inoculated control pine seedlings (Group III, CK) stayed healthy during the experiment, with no dead shoots were observed (Figure 17). In contrast, inoculation with *D. sapinea* alone (Group I) led to the highest shoot mortality (60%) among the inoculated pine twigs after 21 days of inoculation. Necrosis was significantly greater (5–6 mm after 7 days inoculation) in this treatment, compared to co-infection with the potential antagonistic fungus and *D. sapinea.* The 5 pine seedlings in Group II, co-infected with *Pe. fructuariae-cellae* and *D. sapinea,* remained healthy after 7 days of spraying, with an average lesion length of 2 mm, and no dead shoots and yellow needles were observed after 21 days of inoculation.

## 4. Discussion

### 4.1. Endophytic Fungi Detected by Culture-Based Isolation and HTS Method

Endophytic fungal diversity and community composition of different tissues among asymptomatic and symptomatic Mongolian pine were investigated using both culture-based isolation and HTS methods. Our study revealed a broader range of endophytic fungi (1707 species) detected in the HTS data compared to only 57 species isolated through cultivation methods. In contrast to the HTS data, only one species of Basidiomycota fungi was isolated by the culture-based method in this study. This may be partially explained by the fast growth of *D. sapinea* in culture [16,36], which possibly resulted in an under representation of slow-growing fungal species.

Previous studies have shown that HTS can detect a wide range of fungi, including rare and unculturable species, providing insights into fungal community structure and dynamics. Culture-based methods, on the other hand, allow for the isolation of individual strains, which grow rapidly on the provided nutrient medium, facilitating further studies like biocontrol experiments and pathogenicity tests [6,37,38]. Blumenstein et al. [6] indicated that 65% of the species isolated from Scots pine twigs using cultivation methods were detected in the HTS data, while some isolated species were not detected by HTS. In our study, 45% isolated endophytic fungi were obtained from the shoots of the asymptomatic trees (HB), and 43% from the shoots of the symptomatic trees (DB), while in the HTS data, HB exhibiting the highest number of ASVs, but DB with the lowest numbers of ASVs. The selection of primers and databases remains a limiting factor in fully uncovering all species [6]. Therefore, each approach exhibited benefits and limitations. By combining culture-based methods with HTS, a more comprehensive understanding of endophytic fungal communities can be obtained.

Similar to previous studies [4,6,14,39,40], *D.sapinea* was isolated from both asymptomatic and symptomatic pine trees, indicating its endophytic mode. It was the most common endophyte isolated from the symptomatic pine trees and the most common in the HTS data of the symptomatic pine trees. However, its relative abundance varied among various tissues, with *D. sapinea* being most abundant in the current-year shoots of symptomatic pine trees (DB) and less abundant in the shoots and phloem of asymptomatic pine trees (HB & HC). The observations might indicated that *D. sapinea* accumulated mainly in the shoot/twigs and played a role in determine disease development. Bußkamp et al. [4] indicated that during periods of stress such as drought, common endophytes within the Scots pine tree may faced disadvantages, allowing *D. sapinea* to thrive as a main pathogen. It grew into the peridermis and cortex, eventually reaching the vascular tissues in diseased plants, and can quickly occupy more tissues in the host. This could explain why *D. sapinea* is found in higher abundance in the twigs of diseased pines.

In our study, *Sy. polyspora* was isolated from all tissues of both asymptomatic and symptomatic pine trees, with the highest frequency observed in the asymptomatic pine trees. It was also the second most common endophyte isolated from the symptomatic pine trees. *Hormonem*, which includes the species *Sy. polyspora* (formerly known as *Hormonema dematioides* Lagerb. and Melin), was the second most common genus in asymptomatic pine trees according to the HTS data. Additionally, *Sy. polyspora* exhibited the highest isolation frequency in the current-year needles of asymptomatic pine trees (HB), and the lowest frequency in the phloem of symptomatic pine trees (DC). Several studies indicated that *Sy. polyspora*, as a common foliar endephyte of Scots pine, was the most abundant fungus in all disease classes identified with HTS [3,41], and was also the second most common fungus identified by the culture-based method in Blumenstein et al.’s (2021) [18] study. However, *Sy. polyspora* is also known to infect *Pinus pinea* L. in Portugal [42] and *Pinus yunnanensis* Franch. in Southwestern China [43], resulting in symptoms like tip dieback, needles with tan- to yellow-colored lesions, and eventual shoot death.

### 4.2. Endophytic Fungi Community in Different Tissues among Asymptomatic and Symptomatic Mongolian Pine

Alpha diversity analysis unveiled higher species richness and community diversity of endophytic fungi in asymptomatic Mongolian pine compared to symptomatic pine (H vs. D) (Figures S3 and S4). Despite no statistically significant differences in richness indices between asymptomatic and symptomatic trees (Figure S3), significant differences were observed in diversity indices, with the Simpson index (*p* = 0.0012) and Shannon index (*p* = 0.0027) (Figure S4). This implies that although the number of different endophytic fungi may not vary greatly between the two groups, there are notable differences in the composition and evenness of species within the asymptomatic and symptomatic pine. Furthermore, the structure of endophytic fungal communities differed significantly among symptomatic and asymptomatic pine tree. It could be attributed to differences in the presence and abundance of core endophytes. It is possible that the ability of the mycobiome to cause disease symptoms depends on the dominance of certain endophytic fungal species. In other words, the relative abundance and interactions among specific endophytic fungal species may play a crucial role in determining the health status of the pine trees. Differing findings in Scots pine (*P. sylvestris*) were reported by Blumenstein et al. [6], suggesting that diversity indices did not exhibit statistical differences between disease classes, indicating a similar mycobiome in Scots pine of different health classes at the already diseased (Diplodia tip blight) forest site. It appears that host species and tree genotype may influence the composition and community structure of the tree mycobiome [44,45].

The mycobiome of different tissues of asymptomatic and symptomatic pine trees was further analyzed. The lowest richness and community diversity of endophytic fungal communities was observed in the current-year shoots of symptomatic trees (DB). Statistically significant differences were exclusively observed in richness indices among asymptomatic and symptomatic trees in current-year shoots, and there are significant differences in the fungal communities among asymptomatic and symptomatic trees in the needles and shoots. Furthermore, there were no statistically significant differences in richness and community diversity among the three tissues in asymptomatic trees (Figures S5 and S6). Similarly, there were no significant differences in richness among the three tissues were observed in symptomatic tree (Figure S7). However, statistically significant differences in community diversity were observed only between shoots and phloem in symptomatic trees (Figure S8). Furthermore, there are significant differences in community structure related to shoots among asymptomatic and symptomatic trees. These results suggest that the structure and composition of the endophytic fungal community in the specific tissues where typical disease symptoms occur, such as current-year shoots, undergo significant changes. Similar to the study by Kovalchuk et al. [20], they indicated that *Heterobasidion* infection affects the fungal communities in the parts of the tree adjacent to the tissues colonized by the pathogen, with no significant effect on more distant regions.

### 4.3. Antagonism Assay In Vitro and In Vivo

A previous study demonstrated that certain endophytic fungi (*A.alternata*, *Preussia* sp., and *Sy.polyspora*) inhibited the growth of *D. sapinea* in antagonism tests [18]. However, in both our study and in research conducted by Bußkamp [3], *Sy. polyspora* did not exhibit inhibition when cultured alongside *D. sapinea*. This suggests that the observed antagonistic effects of fungal endophytes might be strain specific [8].

In our study, three strains (*Pe. fructuariae-cellae*, *Taifanglania* sp., and *Pa. hawaiiense*) demonstrated inhibitory effects on *D. sapinea*. These strains inhibited *D. sapinea* by over 60%, with *Pe. fructuariae-cellae* exhibiting the highest inhibition rate (73%). Lorenzini et al. [46] discovered that a novel *Penicillium* species from Italy, *Pe. fructuariae-cellae*, which infects grapes but with lower infectivity compared to *Botrytis cinerea*, the primary pathogen. In our study, *Pe. fructuariae-cellae* was assessed as an antagonistic endophytic fungus. Previous research has identified fungi belonging to the genus *Penicillium* as antagonistic endophytes in studies involving other plants. For example, *Penicillium ehrlichii* was isolated from *Camellia sinensis* roots as an antagonistic endophyte against two pathogens, *Colletotrichum gloeosporioides* and *Pseudopestalotiopsis camelliae* [47]. Our findings in vitro and in vivo suggest that *Pe. fructuariae-cellae* holds significant potential for suppressing fungal pathogens of *D. sapinea*. Therefore, future research should focus on extracting and identifying active compounds, as discovering and applying antibacterial agents could aid in the biocontrol of Diplodia tip blight.

## Figures and Tables

**Table 1:** Taxa isolated from asymptomatic and symptomatic Mongolian Pine with the culture-based method.

Isolation Strain No.	Strains	Genbank Accession Number	HA		HB		HC		DA		DB		DC		Total	
			N	IF	N	IF	N	IF	N	IF	N	IF	N	IF	N	IF
EMD1	*Alternaria alternata*	OR002004.1	2	2.86	3	2.78	2	3.51	-	-	2	2.33	5	10.42	14	3.23
EMD2	*Alternaria infectoria*	OR067375.1	-	-	2	1.85	1	1.75	-	-	-	-	-	-	3	0.69
EMD3	*Alternaria seleniiphila*	MK140693.1	-	-	-	-	-	-	-	-	4	4.65	-	-	4	0.92
EMD4	*Alternaria solani*	MN871615.1	-	-	1	0.93	1	1.75	1	1.56	-	-	-	-	3	0.69
EMD5	*Alternaria* sp.	MN096578.1	-	-	-	-	-	-	-	-	1	1.16	3	6.25	4	0.92
EMD6	*Alternaria tenuissima*	KM921667.1	-	-	1	0.93	-	-	1	1.56	-	-	1	2.08	3	0.69
EMD7	*Aspergillus flavus*	MH931826.1	-	-	1	0.93	-	-	5	7.81	4	4.65	2	4.17	12	2.77
EMD8	*Aspergillus* sp.	KR154911.1	-	-	1	0.93	1	1.75	-	-	-	-	-	-	2	0.46
EMD9	*Botryotrichum murorum*	MG770259.1	-	-	1	0.93	-	-	-	-	-	-	-	-	1	0.23
EMD10	*Chaetomium globosum*	MG885806.1	1	1.43	-	-	-	-	-	-	-	-	-	-	1	0.23
EMD11	*Chromolaenicola clematidis*	MT310601.1	-	-	1	0.93	-	-	-	-	-	-	-	-	1	0.23
EMD12	*Cladosporium cladosporioides*	OQ555096.1	-	-	-	-	-	-	3	4.69	-	-	1	2.08	4	0.92
EMD13	*Cladosporium perangustum*	MK722299.1	-	-	-	-	1	1.75	-	-	-	-	-	-	1	0.23
EMD14	*Cladosporium* sp.	MG975642.1	10	14.29	17	15.74	14	24.56	5	7.81	6	6.98	3	6.25	55	12.70
EMD15	*Collariella bostrychodes*	MH931826.1	-	-	1	0.93	-	-	-	-	-	-	-	-	1	0.23
EMD16	*Coprinopsis atramentaria*	KJ817302.1	-	-	1	0.93	1	1.75	-	-	1	1.16	-	-	3	0.69
EMD17	*Curvularia senegalensis*	MT476857.1	-	-	-	-	-	-	-	-	2	2.33	-	-	2	0.46
EMD18	*Deniquelata* sp.	ON705536.1	-	-	-	-	-	-	-	-	1	1.16	-	-	1	0.23
EMD19	*Diplodia sapinea*	MT763348.1	7	10.00	15	13.89	5	8.77	10	15.63	20	23.26	8	16.67	65	15.01
EMD20	*Dothiorella gregaria*	MH791151.1	-	-	1	0.93	-	-	-	-	-	-	-	-	1	0.23
EMD21	*Fusarium fujikuroi*	MH084746.1	-	-	2	1.85	-	-	-	-	-	-	-	-	2	0.46
EMD22	*Fusarium* sp.	OQ422002.1	2	2.86	-	-	-	-	-	-	-	-	-	-	2	0.46
EMD23	*Fusarium verticillioides*	OM956059.1	-	-	4	3.70	-	-	-	-	-	-	-	-	4	0.92
EMD24	*Gregarithecium* sp. *DQD-2016a*	KX364281.1	-	-	2	1.85	1	1.75	-	-	3	3.49	-	-	6	1.39
EMD25	*Iodophanus carneus*	MF161095.1	-	-	1	0.93	-	-	-	-	-	-	-	-	1	0.23
EMD26	*Microsphaeropsis* sp.	MN153956.1	-	-	-	-	-	-	-	-	1	1.16	-	-	1	0.23
EMD27	*Neocamarosporium salicorniicola*	MK809918.1	-	-	-	-	-	-	1	1.56	-	-	-	-	1	0.23
EMD28	*Nothophoma quercina*	ON527450.1	1	1.43	-	-	-	-	-	-	-	-	-	-	1	0.23
EMD29	*Paracamarosporium hawaiiense*	HM751092.1	-	-	1	0.93	-	-	-	-	-	-	-	-	1	0.23
EMD30	*Paraphaeosphaeria* sp.	GU985234.1	-	-	-	-	-	-	2	3.13	-	-	-	-	2	0.46
EMD31	*Penicillium* sp.	MK817609.1	4	5.71	5	4.63	13	22.81	10	15.63	11	12.79	2	4.17	45	10.39
EMD32	*Penicillium fructuariae-cellae*	OP703391.1	-	-	1	0.93	2	3.51	-	-	2	2.33	2	4.17	7	1.62
EMD33	*Penicillium glabrum*	OP681429.1	1	1.43	-	-	-	-	-	-	-	-	-	-	1	0.23
EMD34	*Penicillium oxalicum*	MT597864.1	-	-	-	-	-	-	-	-	1	1.16	-	-	1	0.23
EMD35	*Periconia macrospinosa*	MH345963.1	1	1.43	-	-	-	-	-	-	-	-	-	-	1	0.23
EMD36	*Phialophora cyclaminis*	MW447030.1	-	-	-	-	-	-	-	-	1	1.16	-	-	1	0.23
EMD37	*Phoma* sp.	MT366774.1	-	-	-	-	-	-	2	3.13	-	-	-	-	2	0.46
EMD38	*Podospora australis*	KX015765.1	-	-	-	-	-	-	3	4.69	-	-	-	-	3	0.69
EMD39	*Podospora* sp.	MW349896.1	-	-	-	-	-	-	-	-	-	-	1	2.08	1	0.23
EMD40	*Preussia africana*	OM743863.1	-	-	-	-	-	-	-	-	1	1.16	-	-	1	0.23
EMD41	*Preussia persica*	MW081300.1	-	-	1	0.93	-	-	-	-	-	-	-	-	1	0.23
EMD42	*Preussia* sp.	MT862280.1	-	-	7	6.48	-	-	-	-	-	-	-	-	7	1.62
EMD43	*Rhizopus arrhizus*	ON920727.1	-	-	-	-	-	-	-	-	-	-	3	6.25	3	0.69
EMD44	*Sarocladium zeae*	MZ969671.1	-	-	1	0.93	-	-	-	-	-	-	-	-	1	0.23
EMD45	*Subramaniula cuniculorum*	MT072073.1	-	-	-	-	1	1.75	-	-	2	2.33	-	-	3	0.69
EMD46	*Sydowia polyspora*	MN900630.1	32	45.71	25	23.15	10	17.54	8	12.50	14	16.28	5	10.42	94	21.71
EMD47	*Taifanglania parvispora*	KF719170.1	-	-	-	-	-	-	-	-	4	4.65	-	-	4	0.92
EMD48	*Taifanglania* sp.	KP143100.1	-	-	-	-	-	-	3	4.69	-	-	-	-	3	0.69
EMD49	*Talaromyces amestolkiae*	KT445914.1	3	4.29	-	-	1	1.75	3	4.69	-	-	1	2.08	8	1.85
EMD50	*Talaromyces cecidicola*	MN889416.1	-	-	1	0.93	-	-	-	-	-	-	-	-	1	0.23
EMD51	*Talaromyces funiculosus*	MT367866.1	1	1.43	-	-	-	-	-	-	-	-	-	-	1	0.23
EMD52	*Talaromyces pseudofuniculosus*	OP482383.1	3	4.29	9	8.33	3	5.26	4	6.25	3	3.49	9	18.75	31	7.16
EMD53	*Torula fici*	OW988181.1	-	-	-	-	-	-	-	-	-	-	2	4.17	2	0.46
EMD54	*Tricharina* sp.	MF055701.1	1	1.43	-	-	-	-	-	-	-	-	-	-	1	0.23
EMD55	*Trichoderma afroharzianum*	MT102402.1	-	-	1	0.93	-	-	-	-	-	-	-	-	1	0.23
EMD56	*Trichoderma harzianum*	KY495199.1	-	-	1	0.93	-	-	3	4.69	-	-	-	-	4	0.92
EMD57	*Trichoderma* sp.	MK871263.1	1	1.43	-	-	-	-	-	-	2	2.33	-	-	3	0.69
	Total		70	100.00	108	100.00	57	100.00	64	100.00	86	100.00	48	100.00	433	100.00

“-” means the corresponding endophytic fungal species was not isolated from the specific tissue; “No.” represents the occurrence of certain endophytic fungi in different tissues; the isolation frequency: IF (%) = No. of Isolates/Total No. of Isolates (%).

**Table 2:** Pair_adonis analysis of endophytic fungal community structure at different tissues.

Pairs	R^2^	*p*-Value
HA vs. DA	0.16019719	0.005
HB vs. DB	0.40695618	0.001
HC vs. DC	0.07895605	0.115

**Table 3:** Observations of All Isolated endophytic fungi against *D. sapinea*.

Isolation Strain No.	Strains	Visual Observation
EMD1	*Alternaria alternata*	Pathogen superior
EMD2	*Alternaria infectoria*	Pathogen superior
EMD3	*Alternaria seleniiphila*	Pathogen superior
EMD4	*Alternaria solani*	Pathogen superior
EMD5	*Alternaria* sp.	Pathogen superior
EMD6	*Alternaria tenuissima*	Pathogen superior
EMD7	*Aspergillus flavus*	Equal growth capability
EMD8	*Aspergillus* sp.	Endophyte superior
EMD9	*Botryotrichum murorum*	Pathogen superior
EMD10	*Chaetomium globosum*	Pathogen superior
EMD11	*Chromolaenicola clematidis*	Pathogen superior
EMD12	*Cladosporium cladosporioides*	Pathogen superior
EMD13	*Cladosporium perangustum*	Pathogen superior
EMD14	*Cladosporium* sp.	Pathogen superior
EMD15	*Collariella bostrychodes*	Pathogen superior
EMD16	*Coprinopsis atramentaria*	Pathogen superior
EMD17	*Curvularia senegalensis*	Pathogen superior
EMD18	*Deniquelata* sp.	Pathogen superior
EMD20	*Dothiorella gregaria*	Pathogen superior
EMD21	*Fusarium fujikuroi*	Pathogen superior
EMD22	*Fusarium* sp.	Pathogen superior
EMD23	*Fusarium verticillioides*	Pathogen superior
EMD24	*Gregarithecium* sp. *DQD-2016a*	Pathogen superior
EMD25	*Iodophanus carneus*	Pathogen superior
EMD26	*Microsphaeropsis* sp.	Pathogen superior
EMD27	*Neocamarosporium salicorniicola*	Pathogen superior
EMD28	*Nothophoma quercina*	Pathogen superior
EMD29	*Paracamarosporium hawaiiense*	Inhibition of pathogen growth
EMD30	*Paraphaeosphaeria* sp.	Pathogen superior
EMD31	*Penicillium* sp.	Pathogen superior
EMD32	*Penicillium fructuariae-cellae*	Inhibition of pathogen growth
EMD33	*Penicillium glabrum*	Pathogen superior
EMD34	*Penicillium oxalicum*	Pathogen superior
EMD35	*Periconia macrospinosa*	Pathogen superior
EMD36	*Phialophora cyclaminis*	Pathogen superior
EMD37	*Phoma* sp.	Equal growth capability
EMD38	*Podospora australis*	Pathogen superior
EMD39	*Podospora* sp.	Pathogen superior
EMD40	*Preussia africana*	Pathogen superior
EMD41	*Preussia persica*	Pathogen superior
EMD42	*Preussia* sp.	Pathogen superior
EMD43	*Rhizopus arrhizus*	Pathogen superior
EMD44	*Sarocladium zeae*	Pathogen superior
EMD45	*Subramaniula cuniculorum*	Pathogen superior
EMD46	*Sydowia polyspora*	Pathogen superior
EMD47	*Taifanglania parvispora*	Pathogen superior
EMD48	*Taifanglania* sp.	Inhibition of pathogen growth
EMD49	*Talaromyces amestolkiae*	Pathogen superior
EMD50	*Talaromyces cecidicola*	Pathogen superior
EMD51	*Talaromyces funiculosus*	Pathogen superior
EMD52	*Talaromyces pseudofuniculosus*	Pathogen superior
EMD53	*Torula fici*	Pathogen superior
EMD54	*Tricharina* sp.	Endophyte superior
EMD55	*Trichoderma afroharzianum*	Endophyte superior
EMD56	*Trichoderma harzianum*	Endophyte superior
EMD57	*Trichoderma* sp.	Pathogen superior

**Table 4:** The Inhibition Rate of the Selected Endophytic Fungi against *D. sapinea* after 7 days.

Isolation Strain No.	Strains	Inhibition Rate (%)	Isolated Tissue
EMD48	*Taifanglania* sp.	(61.00 ± 1.73) b	DA
EMD29	*Paracamarosporium hawaiiense*	(72.00. ± 4.00) a	HB
EMD32	*Penicillium fructuariae-cellae*	(73.00. ± 4.62) a	HB and HC

Different letters indicate significant differences (*p* < 0.05).

## Data Availability

Data are contained within the article.

## Supplementary Materials

The following supporting information can be downloaded at: https://www.mdpi.com/article/10.3390/jof10030212/s1. Figure S1: ANOSIM analysis of endophytic fungal communities from symptomatic and asymptomatic trees. Figure S2: ANOSIM analysis of endophytic fungal communities in A (needles), B (shoots), and C (phloem) samples. Figure S3: Box plots of ACE and Chao1 indices for samples of symptomatic and asymptomatic pine. Figure S4: Boxplots of Simpson and Shannon indices for samples of symptomatic and asymptomatic pine. Figure S5: Box plots of ACE and Chao1 indices for needles, shoots and phloem samples of asymptomatic pine. Figure S6: Box plots of Simpson and Shannon indices for needles, shoots and phloem samples of asymptomatic pine. Figure S7: Box plots of ACE and Chao1 indices for needles, shoots and phloem samples of symptomatic pine. Figure S8: Box plots of Simpson and Shannon indices for needles, shoots and phloem samples of symptomatic pine.
