# Supplementary material for: Diversity, Community Structure, and Antagonism of Endophytic Fungi from Asymptomatic and Symptomatic Mongolian Pine Trees
Source: J Fungi (Basel). 2024 Mar 13;10(3):212. doi: 10.3390/jof10030212 (PMC10971010; doi:10.3390/jof10030212)
Supplement: Supplementary file 1 [file jof-10-00212-s001.zip › Figure S1-Figure S8.pdf]

Figure S1: ANOSIM analysis of endophytic fungal communities from symptomatic and asymptomatic trees.
Figure S2: ANOSIM analysis of endophytic fungal communities in A (needles), B (shoots), and C (phloem) samples.
FIgure S3: Box plots of ACE and Chao1 indices for samples of symptomatic and asymptomatic pine.
Figure S4: Boxplots of Simpson and Shannon indices for samples of symptomatic and asymptomatic pine.
Figure S5: Box plots of ACE and Chao1 indices for needles, shoots and phloem samples of asymptomatic pine.
Figure S6: Box plots of Simpson and Shannon indices for needles, shoots and phloem samples of asymptomatic pine.
Figure S7: Box plots of ACE and Chao1 indices for needles, shoots and phloem samples of symptomatic pine.
Figure S8: Box plots of Simpson and Shannon indices for needles, shoots and phloem samples of symptomatic pine.
