# Supplementary figures and images for: Diversity, Community Structure, and Antagonism of Endophytic Fungi from Asymptomatic and Symptomatic Mongolian Pine Trees
Source: J Fungi (Basel). 2024 Mar 13;10(3):212. doi: 10.3390/jof10030212 (PMC10971010; doi:10.3390/jof10030212)

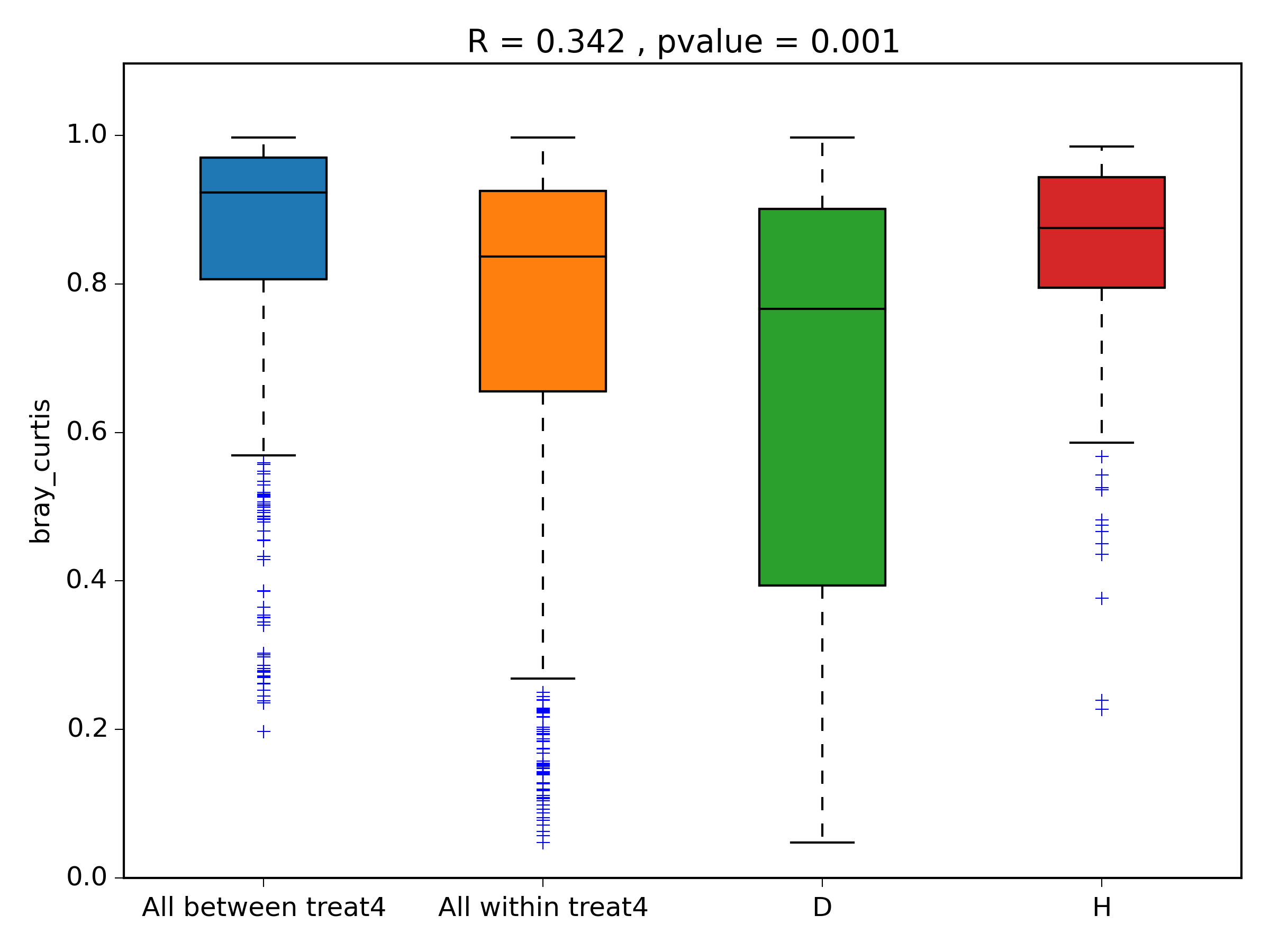

Supplement: Supplementary file 1 [file jof-10-00212-s001.zip › Figure S1.png]

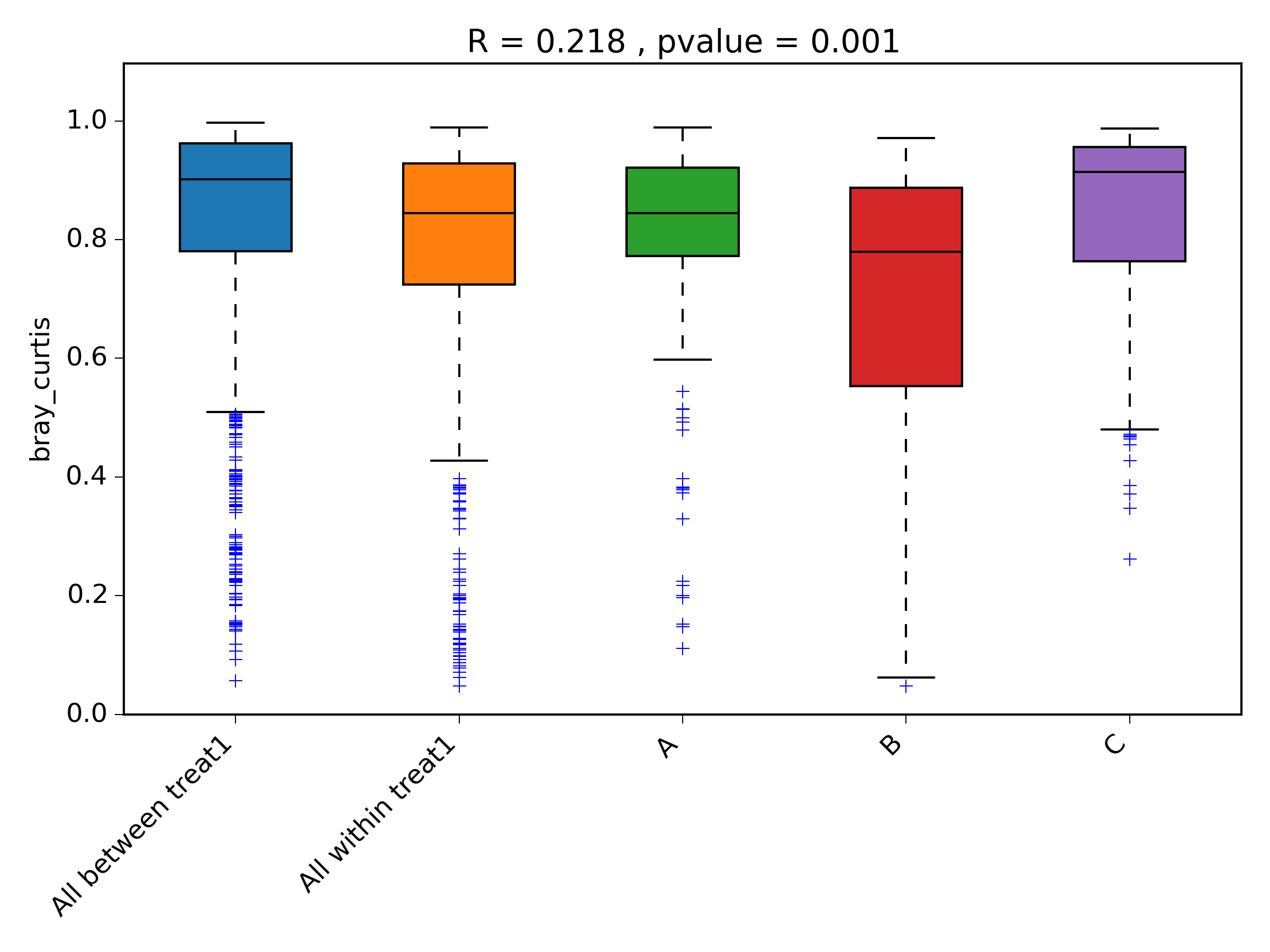

Supplement: Supplementary file 1 [file jof-10-00212-s001.zip › Figure S2.png]

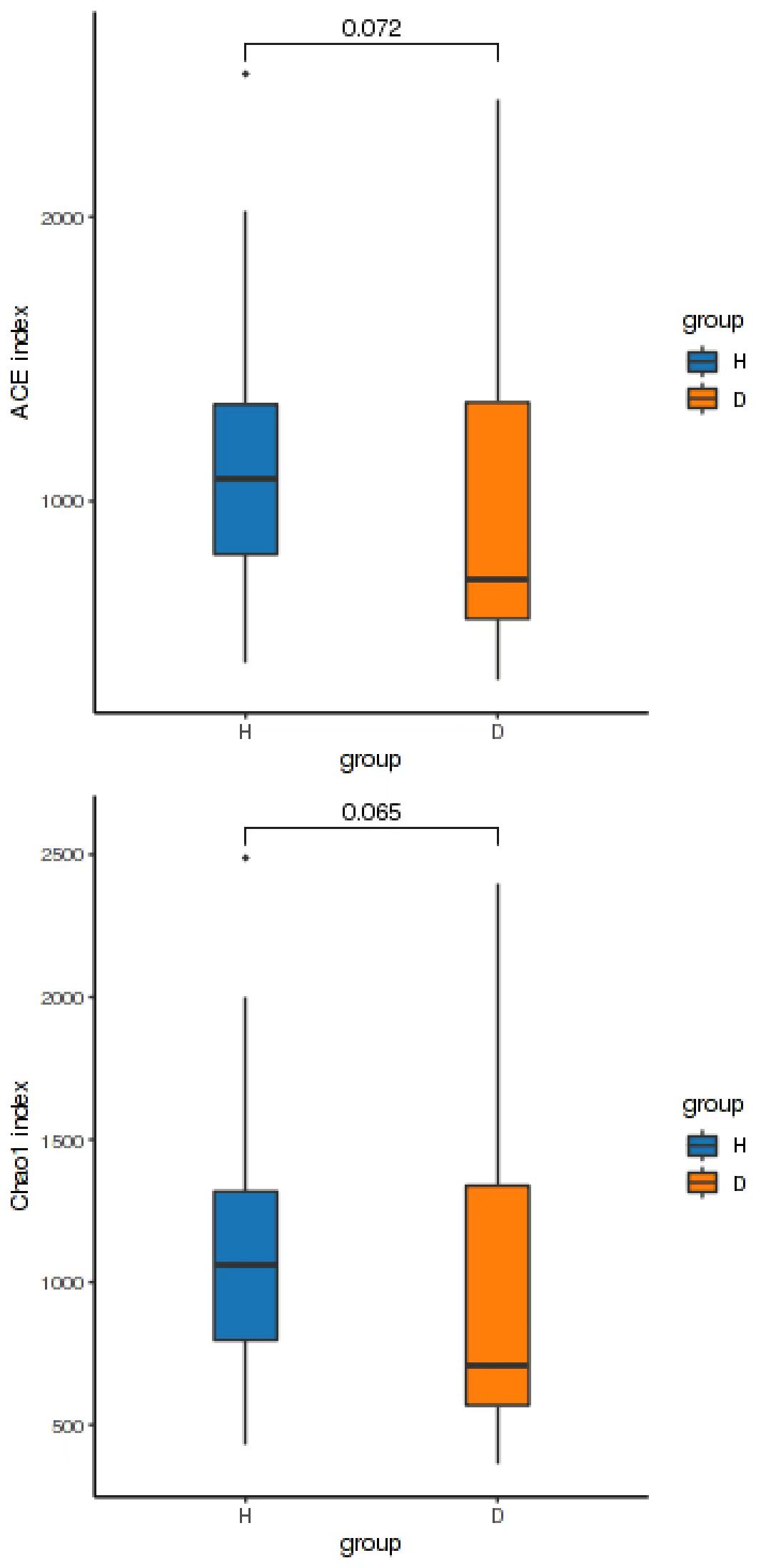

Supplement: Supplementary file 1 [file jof-10-00212-s001.zip › Figure S3.jpg]

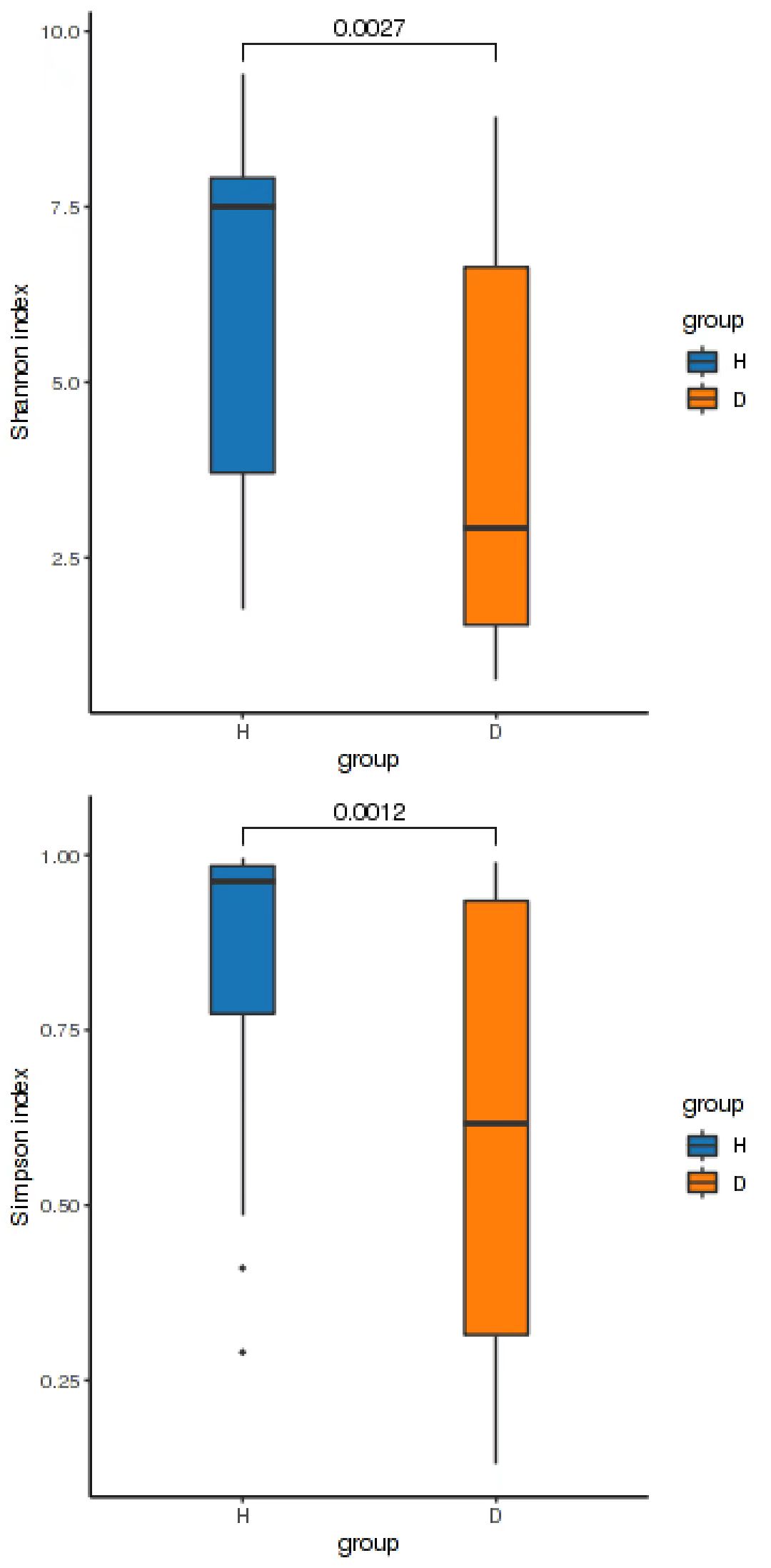

Supplement: Supplementary file 1 [file jof-10-00212-s001.zip › Figure S4.jpg]

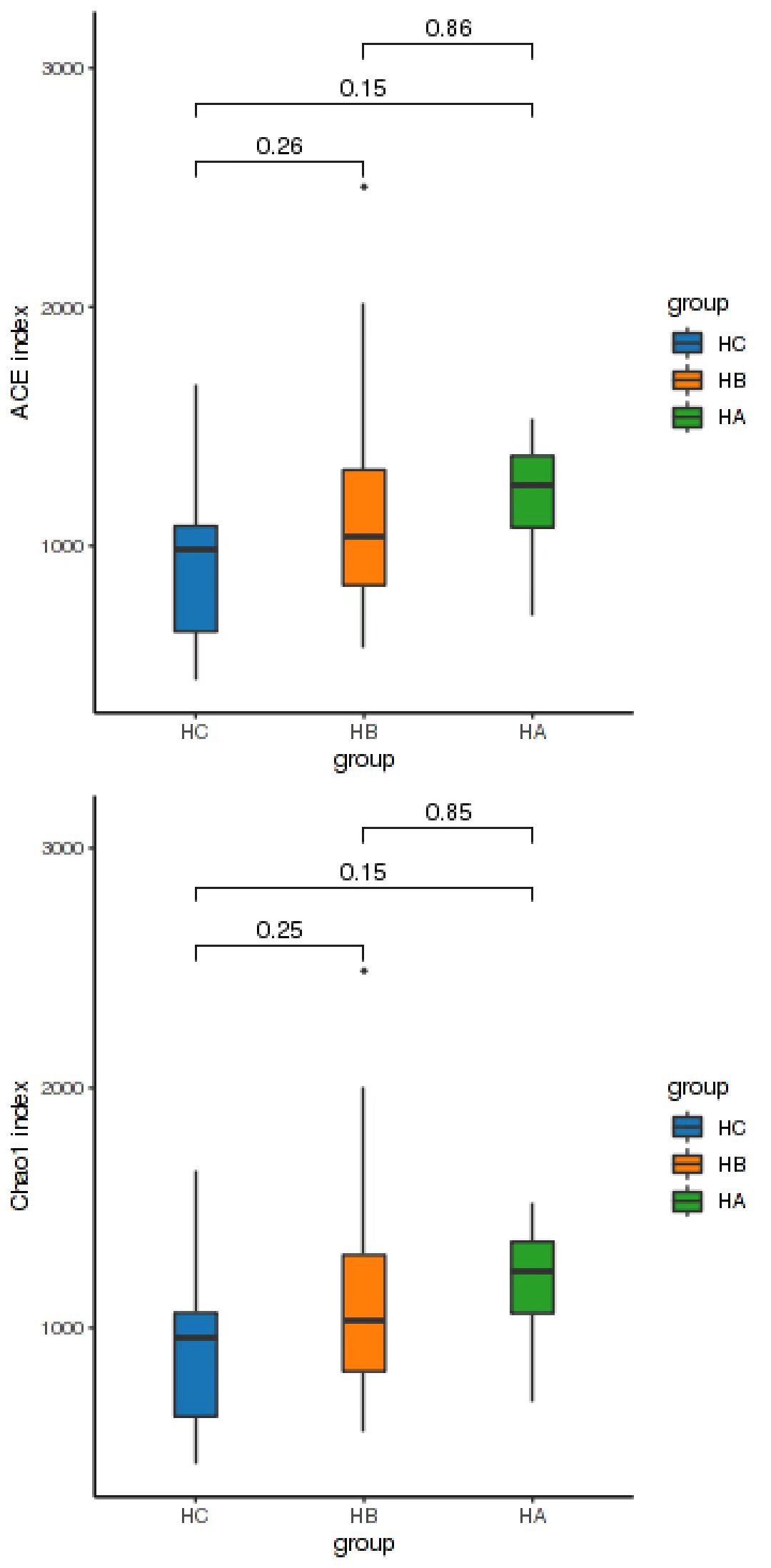

Supplement: Supplementary file 1 [file jof-10-00212-s001.zip › Figure S5.jpg]

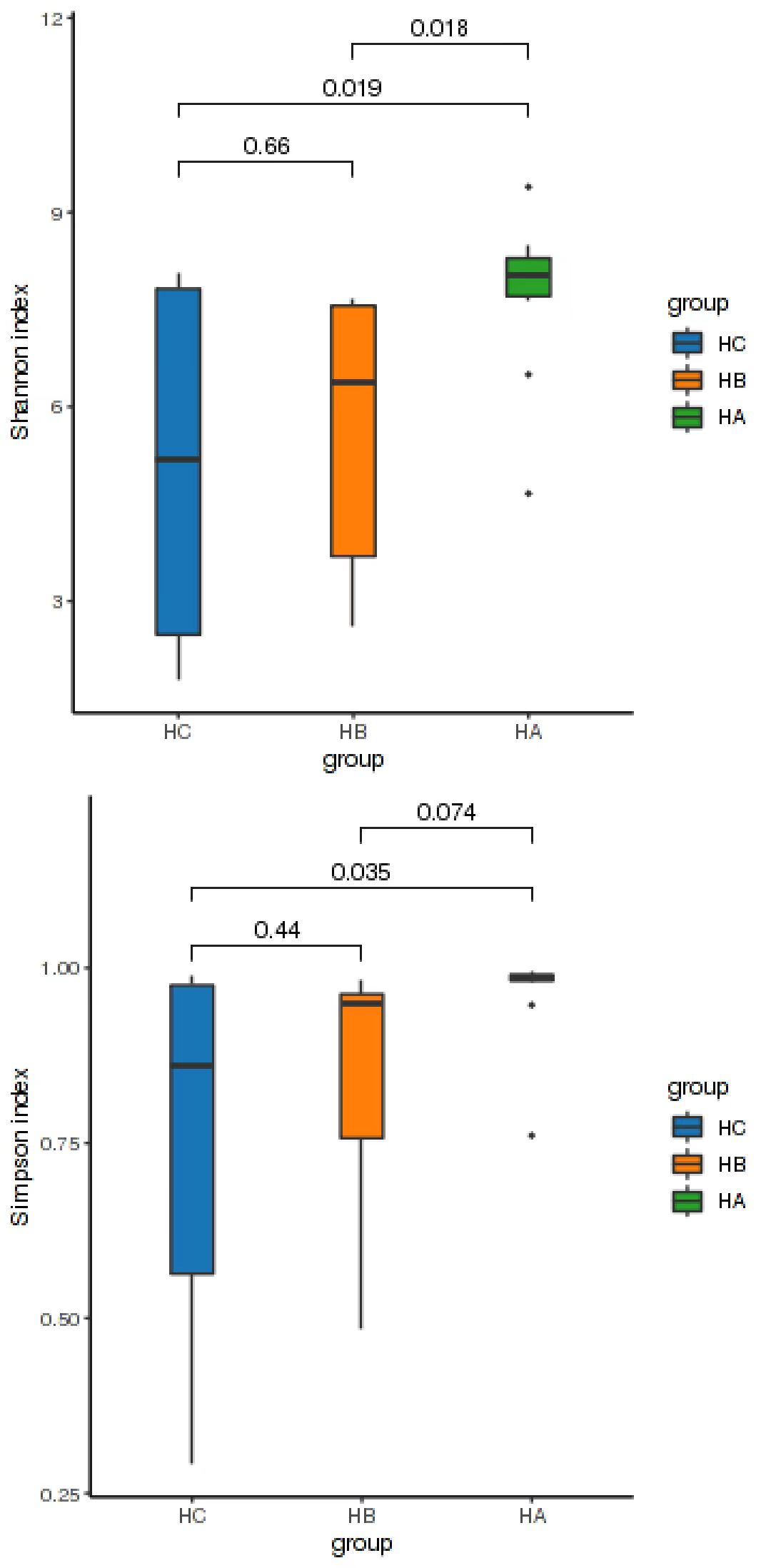

Supplement: Supplementary file 1 [file jof-10-00212-s001.zip › Figure S6.jpg]

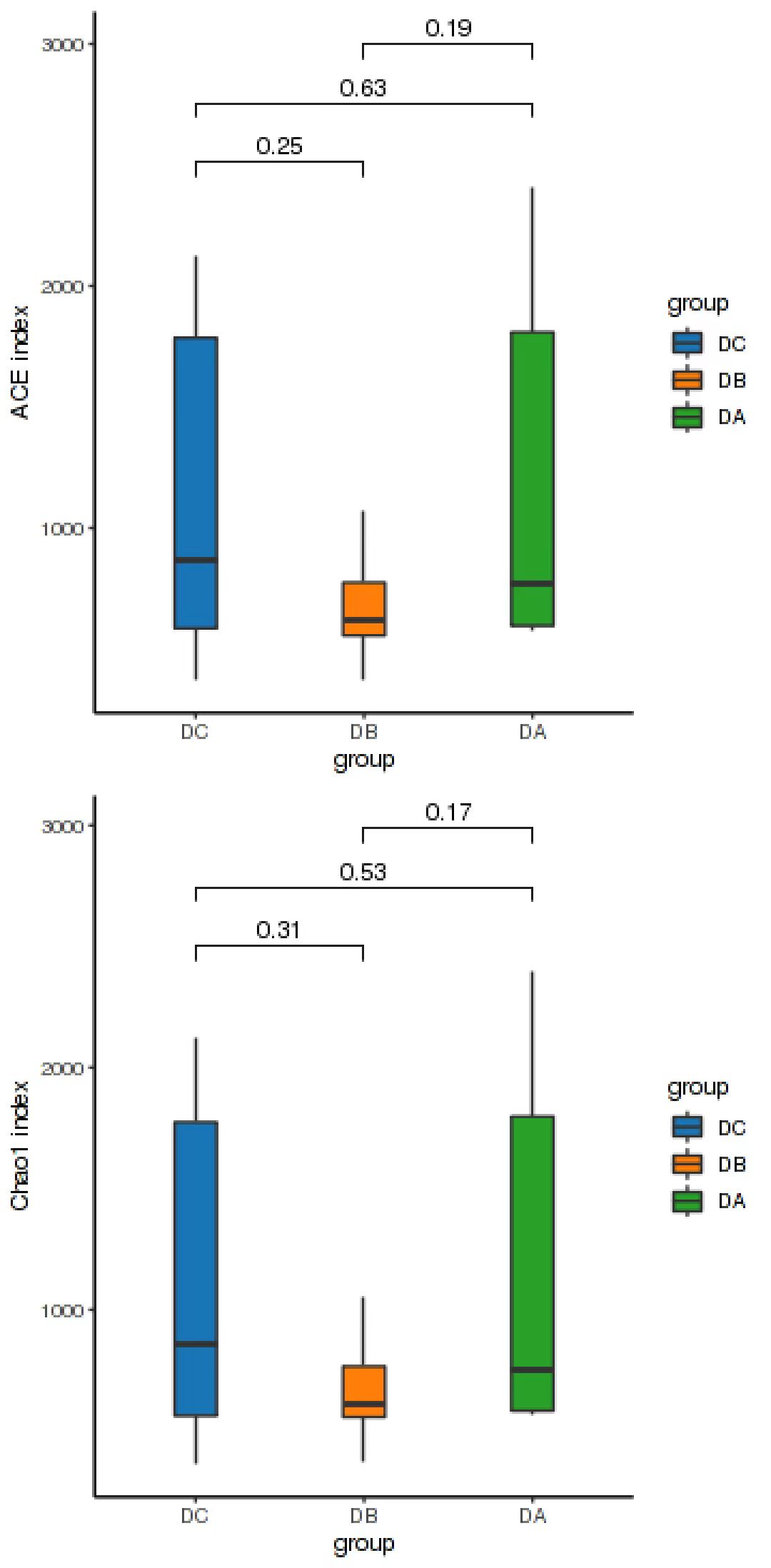

Supplement: Supplementary file 1 [file jof-10-00212-s001.zip › FigureS7.jpg]

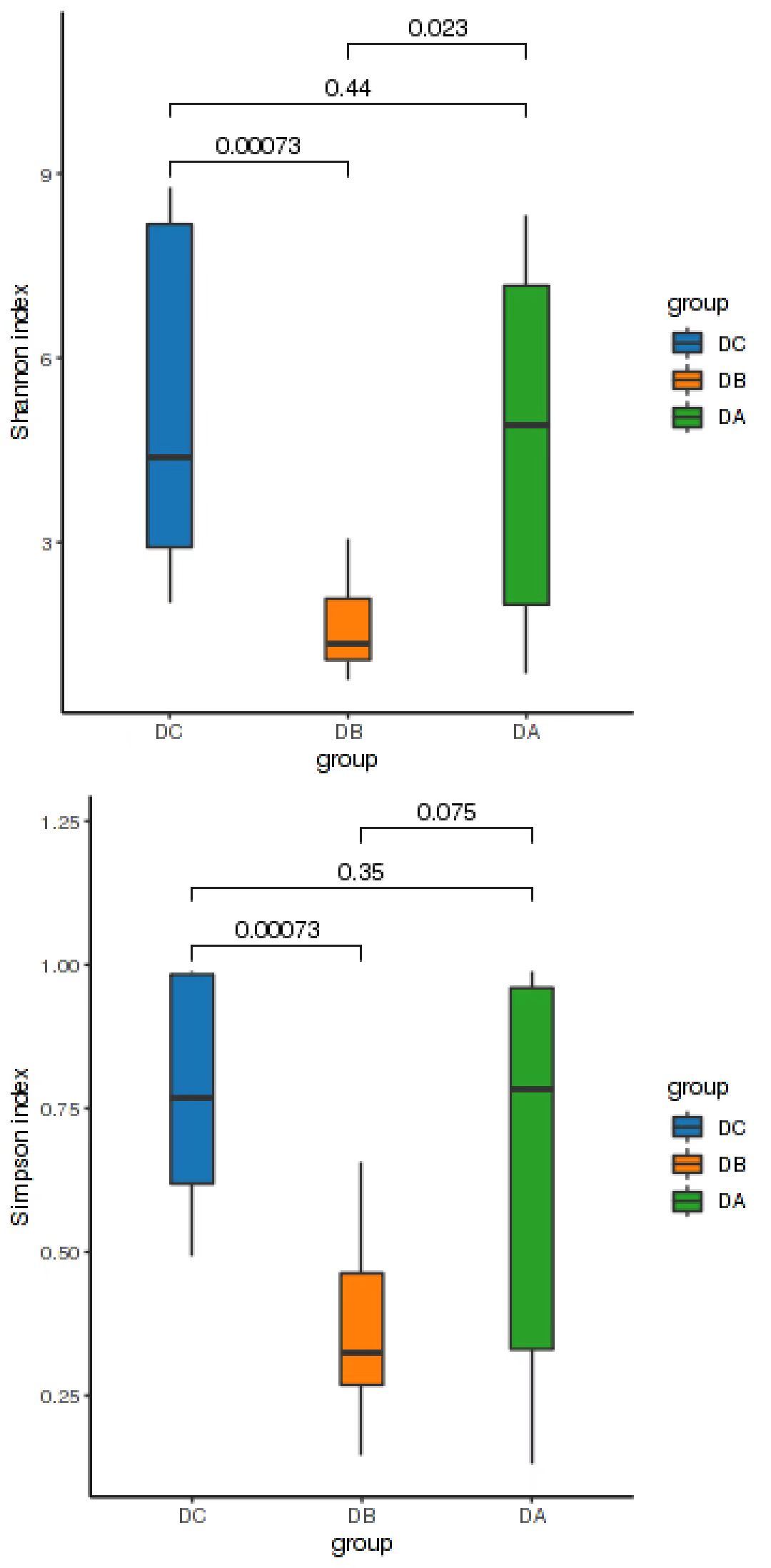

Supplement: Supplementary file 1 [file jof-10-00212-s001.zip › FigureS8.jpg]
